# The *msf* gene causes condition-specific shifts in global gene expression in *Haemophilus influenzae*

**DOI:** 10.1128/mbio.01263-26

**Published:** 2026-07-29

**Authors:** Evangeline M. Williams, Mary C. Marino, Jocelyn Hammond, Laura Anastor-Walters, Bhaswati Sen, Kalisse Horne, Azad Ahmed, Steven Lang, Sergey Balashov, Benjamin Janto, Garth D. Ehrlich, Joshua Chang Mell

**Affiliations:** 1 Center for Genomic Sciences, Institute for Molecular Medicine and Infectious Disease and Department of Microbiology & Immunology, Drexel University College of Medicine 12312, Philadelphia, Pennsylvania, USA; 2 Department of Otolaryngology–Head & Neck Surgery, Drexel University College of Medicine 12312, Philadelphia, Pennsylvania, USA; University of California, Berkeley, Berkeley, California, USA

**Keywords:** *Haemophilus influenzae*, transcriptome, yeast two-hybrid, Sel1-like repeat, otitis media, biofilms, host-pathogen interactions, outer membrane proteins, adhesins

## Abstract

*Haemophilus influenzae* is a diverse human-restricted bacterium that normally colonizes the healthy nasopharynx but also causes common infections. Comparisons of clinical isolate genomes previously identified a gene, *msf*, that contained Sel1-like repeats that were associated with clinical disease. Mutant analysis had further found that *msf* improved survival in macrophages and increased systemic infection in an animal model. However, the role of *msf* in other conditions and its molecular function remain unknown. To identify protein-protein interactions with Msf, a yeast two-hybrid screen against an *H. influenzae* prey library was conducted, which found potential interactions with lipoprotein exporter protein LolD and an autotransporter adhesin Hap. To identify effects of *msf* on gene expression, we compared wild-type and mutant strains grown in multiple culture conditions by RNA-seq. The results indicate that *msf* modulates global gene expression in a condition-dependent manner, exerting an especially strong influence in starved surface-attached biofilm cells. The few consistent changes in mutants’ planktonic exponential and stationary phases included decreased expression of two paralogous autotransporter adhesins. By contrast, mutant cells in starved surface-attached biofilms had dramatic changes in expression, including upregulation of protein translation and downregulation of alternative carbon metabolism. However, assays of 24 hour biofilm phenotypes found only subtle gene expression changes. Together, the results point to a speculative model of Msf functioning as an envelope-associated chaperone whose presence affects the relative expression of proteins at the outer membrane.

**IMPORTANCE:**

Comparing genomes from different clinical isolates of the same pathogenic bacterial species has identified genes associated with virulence, but many of these are understudied or have no known function. The *msf* gene was previously implicated as a virulence factor in *Haemophilus influenzae*, a common cause of mucosal diseases including middle-ear and chronic lung infections. This study finds that the *msf* gene causes condition-specific changes in gene expression, with especially dramatic changes in starved surface-attached biofilm cells. Along with identification of putative protein-protein interaction partners, the results provide new clues as to the molecular and cellular function of Msf, potentially as an envelope-associated chaperone involved in membrane protein trafficking. Understanding how virulence-associated genes like *msf* modulate bacterial responses to the environment may help explain why some bacterial strains remain harmless colonizers while others become pathogens.

## INTRODUCTION

Many bacteria colonize hosts without causing disease but become pathogenic due to complex interactions between host genetics, environmental factors, co-infections, immune responses, and within-species bacterial genetic diversity (1–5). In addition to allelic variation, many species also have extensive diversity in gene content (3, 5–7). Correlating gene content with clinical outcomes through genome-wide association studies (GWAS) can identify virulence genes and provide insights into mechanisms of pathogenesis (8–11).

*Haemophilus influenzae* is a human-restricted bacterium prevalent in healthy children’s nasopharynxes (12, 13). However, it is also a major cause of morbidity due to its role in mucosal infections including sinusitis, conjunctivitis, middle ear infections (otitis media) (14–19), and lung infections (especially in chronic obstructive pulmonary disease) (20–22), as well as now-rarer invasive diseases like bacteremia and meningitis (23–26). Natural variation in gene content is known to play a pivotal role in determining *H. influenzae* pathogenicity (4, 5, 27–31). *H. influenzae* is useful for understanding commensal-to-pathogen transitions because of its role in common diseases, prevalence in healthy humans, experimental tractability, extensive genomic diversity, and traits like natural competence and phase variability that accelerate in-host evolutionary adaptation (2–5, 30, 31).

In a previous GWAS of *H. influenzae*, disease-associated genes were identified (29, 32), including a gene family encoding proteins with Sel1-like repeats (SLRs). SLRs are found in many proteins across all domains of life and are known to form solenoid structures related to the tetratricopeptide (TPR) motif (33). SLRs are involved in myriad processes, including mediating interactions between membranes, protein-protein interactions, and signal transduction. Some bacterial SLRs are known to mediate host-pathogen interactions, as in *Helicobacter pylori* and *Legionella pneumophila* (33–36). *H. influenzae* has a core SLR gene and “distributed” or “accessory” SLR genes with variable numbers of repeats. All *H. influenzae* SLR proteins carry an N-terminal SP1-type signal peptide, suggesting transport via the Sec system through the inner membrane (IM) to the periplasm or beyond, as well as a short conserved C-terminus of unknown function (32). The association of SLR genes with clinical disease prompted further investigation into their role.

Mutant analysis found that deleting four tandem copies from the PittII strain (an otorrhea isolate) resulted in survival defects after uptake by macrophages, as well as decreased systemic dissemination in a chinchilla model, leading to the gene name *msf* for “macrophage survival factor” (32). Knockout of *msf* in another clinical isolate, 86-028NP, and knock-in into non-pathogenic lab strain Rd KW20 supported these conclusions, and microarray analysis found altered expression of genes associated with oxygen sensing (5, 37). Since bacteria within host cells and biofilms are key contributors to persistent infections (38), with both experiencing low nutrients and oxygen, we reasoned that *msf* might play a role in biofilms. Here, we sought to gain insights into *msf* with a yeast two-hybrid (Y2H) protein-protein interaction screen and an RNA-seq experiment comparing wild-type and mutant strains grown in multiple culture conditions.

## RESULTS

The *Haemophilus influenzae* gene *msf* is enriched in disease and implicated in improved intracellular survival; however, little else is known about its function (29, 32). Thus, we (i) applied yeast two-hybrid screening to identify putative interactions of Msf with other proteins; (ii) used RNA-seq analysis of wild-type and mutant strains; and (iii) assayed wild-type and mutant strains for changes in biofilm biomass, viability, and metabolic activity.

### Putative interactions of Msf with cell envelope proteins

**Fig 1:**
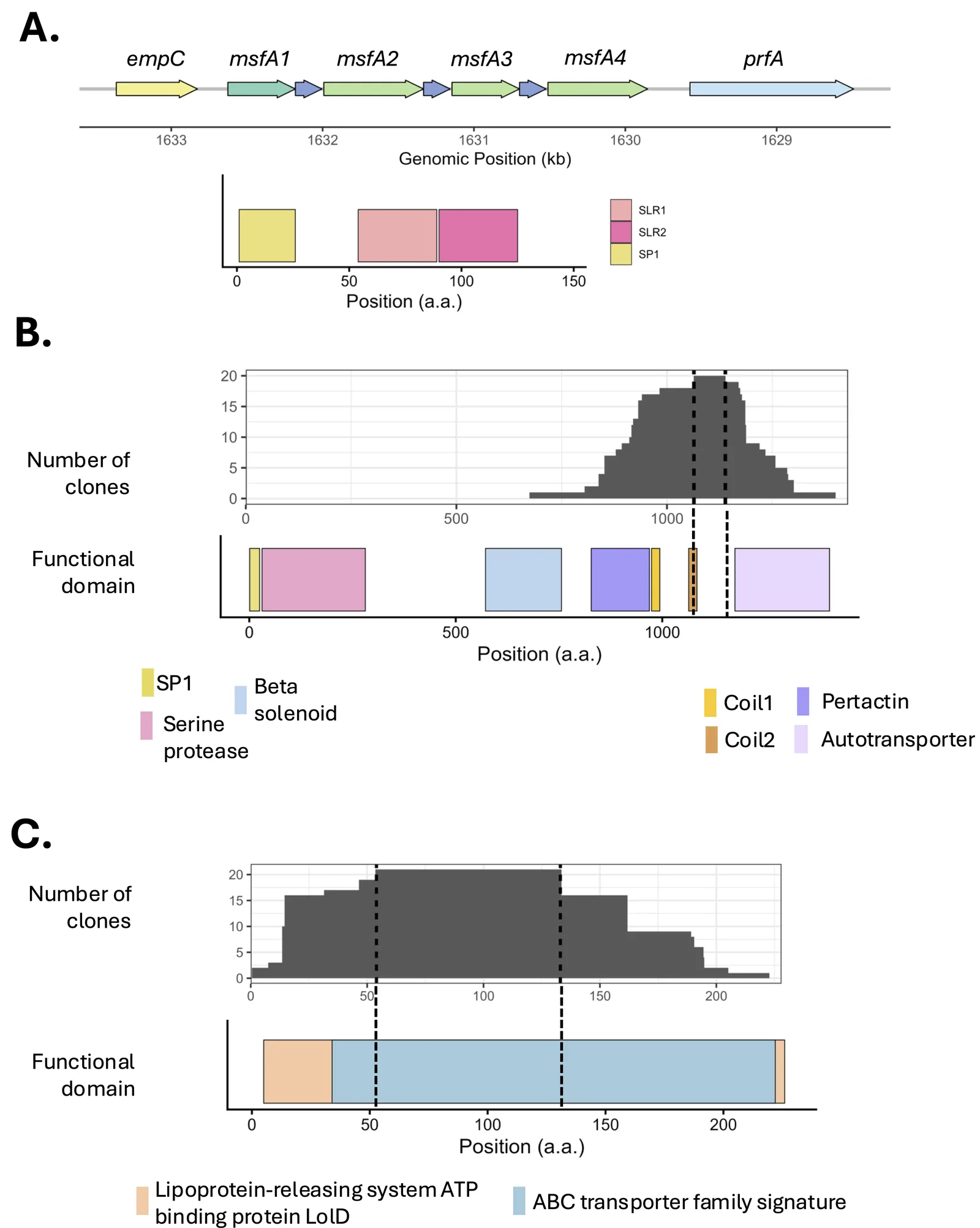
Localization of putative Msf protein-protein interactions with LolD and Hap. (**A**) (Top) Genomic organization of the PittII *msf* locus, which contains four tandem repeats of SLR-containing genes between *epmC* (elongation factor P hydroxylase) and *prfA* (protein chain release factor RF1). Each *msf* gene contains a signal peptide (SP1) and variable SLR repeats (bottom). Three very short hypothetical ORFs interspersed among *msf* copies are also shown. For (**B**) LolD and (**C**) Hap, the coverage of high-quality clone hits (an “A” PBS score with 100% identity and query coverage) across each protein was used to narrow the interacting region of the bait. Horizontal lines indicate the minimum spanning interval where the interaction with Msf is predicted to occur. Signal peptide prediction results using SignalP are shown in Fig. S2.

Since *msf* encodes an N-terminal signal peptide and other bacterial SLRs mediate host-cell interactions (32, 33), we hypothesized that Msf interacts with host proteins. To identify interaction partners, we used an *msfA1* clone (the first ORF at the four-copy *msf* locus in PittII; Fig. 1A) as the bait in a commercial Y2H screen against a prey cDNA library generated from human monocyte-derived THP-1 cells (39, 40); however, no high-confidence interactions were detected. Although interactions with host proteins were not excluded, we next tested for interactions of Msf interacts with bacterial proteins. A prey library was produced from genomic DNA of 11 diverse *H. influenzae* isolates and screened against the *msfA1* bait. Prey fragments from 295 clones positive for interactions with *msf* were sequenced (39, 40). To identify prey interaction partners, 284 in-frame prey sequences were aligned to a database of *H. influenzae* genes (Table S1). Of these, 95 in-frame prey fragments were contained within 19 single genes, that is, with 100% query coverage, since some fragments spanned multiple ORFs (Table 1; Table S2). Only two interactions had high confidence and were supported by >10 independent prey fragments (Table 1, rows 1 and 2), both of which are localized to the cell envelope (Fig. 1B and C). Only two proteins had high-confidence interactions with Msf: (i) LolD with 21 high-confidence prey fragments is the cytoplasmic ATPase subunit of the inner membrane LolCDE transporter that traffics lipoproteins to the outer membrane (OM) (41–44). The minimum spanning interval of LolD that interacts with Msf suggests that Msf may interact with the ATP-binding cassette (Fig. 1B), though a LolD-Msf interaction is incompatible with the signal peptide on Msf. (ii) The Hap protein (18 high-confidence prey fragments) is an outer membrane autotransporter adhesin (45–48). Like Msf, Hap contains an N-terminal SP1 signal peptide for Sec-dependent export. Fragment mapping suggests that Msf may interact with Hap near the autotransporter domain that anchors the matured protein into the outer membrane (Fig. 1B). These results provide evidence for interactions of Msf with LolD and Hap. Although these two proteins have distinct cellular localizations, they are consistent with a role for Msf at the cell envelope.

**TABLE 1:** MsfA1 protein-protein interactions detected by yeast two-hybrid^*a*^

Gene cluster	Predicted function	A	B	C	D	Total	Note
*lolD*	Lipoprotein-releasing system ATP-binding protein LolD	21	0	0	0	21	Cytoplasm at inner membrane; LolABCDE complex
*hap*	Adhesion and penetration protein Hap	18	0	0	2	20	Outer membrane; signal peptide, transmembrane, and coiled-coil
*lysS*	Lysine—tRNA ligase	0	6	0	5	11	Cytoplasm; coiled-coil
HindIR	Type I restriction enzyme HindI R protein	0	4	0	0	4	Likely cytoplasmic
*purC*	Phosphoribosylaminoimidazole-succinocarboxamide synthase	0	4	0	0	4	Likely cytoplasmic
*rpoD*	RNA polymerase sigma factor RpoD	0	3	2	6	11	Cytoplasmic
Group_9397	Hypothetical protein	0	0	2	0	2	
*proS*	Prolyl-tRNA synthetase	0	0	0	7	7	Cytoplasmic
*ttgA*	Putative efflux pump periplasmic linker ttgA	0	0	0	3	3	Periplasm; signal peptide
*ispB*	Octaprenyl-diphosphate synthase	0	0	0	2	2	Cytoplasmic
*mukB*	Structural maintenance of chromosome-related protein	0	0	0	2	2	Cytoplasmic
*rpoC*	RNA polymerase beta-prime subunit	0	0	0	2	2	Cytoplasmic
Group_4741	Non-canonical purine NTP pyrophosphatase	0	0	0	1	1	
Group_7228	Integrating conjugative element PFGI_1 class ParB family protein	0	0	0	1	1	Envelope-associated
Group_7793	Putative terminase ATPase subunit	0	0	0	1	1	
*gyrA*	DNA gyrase A subunit	0	0	0	1	1	Cytoplasmic
S.HinC486III	Type I restriction-modification system specificity determinant subunit S	0	0	0	1	1	Likely cytoplasmic
*priA*	Primosomal protein N'	0	0	0	1	1	Cytoplasmic

^*a*^ A yeast two-hybrid (Y2H) screen with *msf* bait yielded 284 in-frame prey clones, of which 95 prey fragments mapped to 19 single protein-coding gene clusters with 100% identity and 100% query coverage. The count of prey fragments with predicted biological score (PBS) A through D were further used to rank interactions. PBS values range from A (high-confidence interactions supported by multiple independent fragments) to D (interactions supported by only a single fragment), while F denotes known artifactual interactions. A full summary of results including prey fragments with segments from more than one gene are in Table S2.

### RNA-seq transcriptome profiling of wild type and Δ*msf* in multiple *in vitro* conditions

To gain insights into the consequences of expressing *msf*, we performed RNA-seq across multiple growth conditions. The PittII isolate served as wild type, and the Δ*msf* mutant lacks the whole *msf* locus (Fig. 1A) (32). Wild type and mutant were grown in eight conditions in triplicate (Table 2). RNA was extracted, converted to ribosomal RNA (rRNA)-depleted sequence libraries, and pooled for sequencing with a target of ~1 million reads per sample. Reads were aligned to a complete reference, and gene-level count tables were generated. Quality metrics are summarized in Table S3; counts with gene annotations are in Table S4. These data provide a compendium of wild-type and Δ*msf* global transcriptomes across a range of *in vitro* conditions.

**TABLE 2:** Overview of experimental conditions used for RNA-seq^*a*^

Condition	Name, abbreviation	Culturing condition
Shaking	Logarithmic, Log	Aerobic mid-log phase in sBHI
	Stationary, Sta	Aerobic stationary phase in sBHI
Static	Supernatant, Sup	Free-floating supernatants from 24 hour static sBHI cultures
	Well-associated, biofilm, Bio	Well-associated biofilms from 24 hour static sBHI cultures
Shaking, planktonic	Anaerobic early, Ane Early	Anaerobic early-log phase in sBHI
	Anaerobic late, Ane Late	Anaerobic late-log phase in sBHI
	CDM^*b*^ logarithmic, CDM Log	Aerobic mid-log phase in CDM
	CDM stationary, CDM Sta	Aerobic stationary phase in CDM

^*a*^ Summary table of the eight conditions for which RNA-seq was performed. Four key conditions are emphasized: exponential and stationary phase cells from aerated shaking cultures, and two fractions from 24 hour static cultures—free-floating supernatant cells and well-associated biofilm cells. Additional conditions are described in Fig. S1. The conditions capture differences in growth phase, nutrient availability, oxygenation, and stress.

^*b*^ CDM, chemically defined medium.

### The *msf* accessory gene has condition-dependent effects on gene expression

**Fig 2:**
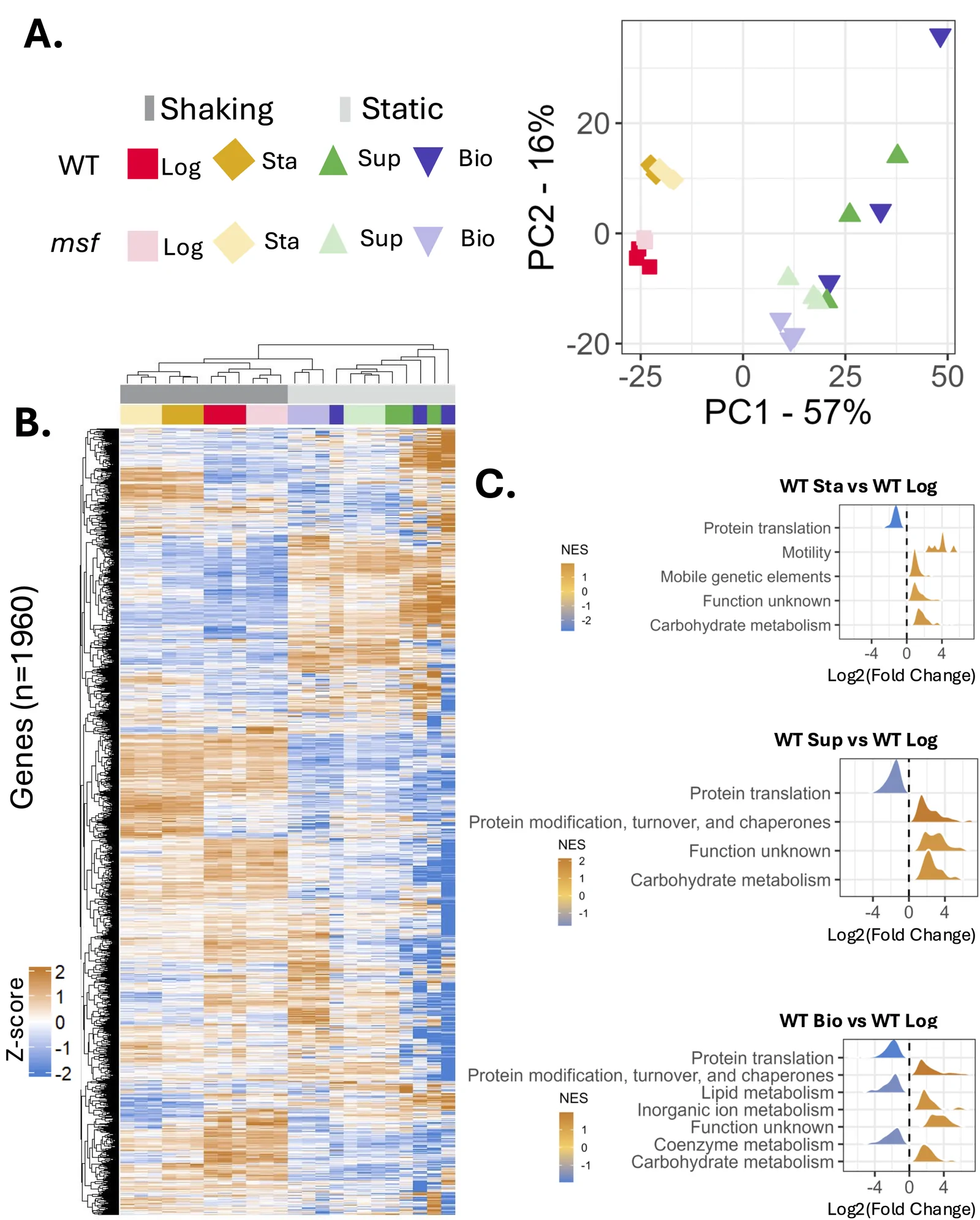
Transcriptome differences between Δ*msf* and wild type among conditions. (**A**) PCA of VST-normalized counts using the 500 highest-variance genes. Genotypes are shown with filled (wild type) or open (Δ*msf*) symbols. Color and shape represent condition: red squares for log, yellow diamonds for stationary, green triangles for supernatants, and purple inverted triangles for well-associated. (**B**) Heatmap of Z-scored VST-normalized counts for 1,960 PittII genes (rows) across five conditions in triplicate (columns). Orange indicates increased relative transcript abundance; blue indicates decreased. Lower sidebar colors follow (**A**), with the darker shade indicating wild type and the lighter shade Δ*msf*. The upper sidebar indicates shaking (dark gray) or static (light gray) cultures. (**C**) GSEA of COG categories comparing each low-nutrient condition to log phase, shown as ridge plots of the “leading edge” genes in the COG category that most contributed to enrichment. The color represents the normalized enrichment score (NES) of each COG category.

To visualize differences in gene expression among samples, a principal component analysis (PCA) was performed on RNA-seq counts after variance-stabilizing transformation (VST; which roughly log2-scales normalized counts [49]), (Fig. 2A; Fig. S1). Transcriptomes were strongly differentiated by condition, indicating the distinct physiological states of the cells. Cells collected from 24 hour static cultures—both free-floating supernatants and well-associated biofilms—were highly differentiated from the other conditions (i.e., PC1 explained 57% of the variation). However, wild-type supernatant and biofilm transcriptomes were poorly resolved, suggesting that these cells are in a similar state, irrespective of whether surface attached. These results are consistent with cells in both fractions having low nutrient and oxygen availability.

The effects of genotype were distinct between shaking and static cultures. Transcriptomes were not as distinct between genotypes in shaking cultures (Fig. 2A). Additional tested conditions, including growth anaerobically or in chemically defined media, showed little difference by genotype (Fig. S1 and S4). Specifically, anaerobic cultures had only 12 and 1 differentially expressed genes (DEGs) for two anaerobic culture conditions (PCA in Fig. S1; pairwise DEG counts in Table S5; gene lists in Table S6). By contrast, the static cultures showed a substantial separation between genotypes (Fig. 2A): supernatant and well-associated fractions formed distinct clusters in Δ*msf*, in contrast to wild type.

Consistent with the PCA, expression differences among samples for all genes are shown as a heatmap in Fig. 2B. Hierarchical clustering first separated shaking from static cultures. Within each shaking condition, samples subclustered by genotype. By contrast, in 24 hour static cultures, wild-type and Δ*msf* transcriptomes had large differences, whereas supernatant and well-associated cells were less differentiated. Also consistent were DEG counts seen between conditions for each genotype (Table 3; full pairwise results in Table S5). Hundreds were identified between most conditions, with more when comparing between shaking and static conditions. DEG counts for most comparisons were similar between genotypes, except one strong outlier between the two 24 hour static culture conditions: in wild type only 7 DEGs had >2-fold change, in sharp contrast to the 247 seen in the mutant (Table 3). These results show that *msf* has condition-specific effects on gene expression and pronounced effects in starved 24 hour static culture biofilms.

**TABLE 3:** DEGs between pairs of conditions for each genotype^*a*^

Genotype	Treatment		Control		
		Exponential	Stationary	Supernatant	Well-associated
WT	Exponential	–^*b*^	212/274	393/423	418/360
	Stationary	274/212	–	362/345	353/320
	Supernatant	423/393	345/362	–	4/3
	Well-associated	360/418	320/353	3/4	–
Δ*msf*	Exponential	–	269/310	431/432	443/432
	Stationary	310/269	–	386/287	428/287
	Supernatant	432/431	287/386	–	92/155
	Well-associated	432/443	287/428	155/92	–

^*a*^ DEGs were determined by pairwise Wald tests using DESeq2 with 1,960 genes from PittII. The two slash-separated numbers are for up- and downregulated DEGs, respectively, using as thresholds an FDR-corrected *P*-value <0.05 and |log_2_(FC)| >1. Shown are **c**omparisons within wild-type PittII and within the Δ*msf* mutant.

^*b*^ – indicates self pairwise comparisons, which were excluded from the analysis.

### Large-scale differences in expression of COG categories among conditions

To provide a context for how the effects of Δ*msf* vary among conditions, we first present an analysis of the large-scale pathway changes between conditions in wild type. To label protein-coding genes with functional categories, we used eggNOG-mapper (50), identifying “cluster of orthologous genes” (COG) IDs for 1,877 of 1,906 protein-coding genes and a functional COG category for 1,517 of 1,906 protein-coding genes (51). We next used gene set enrichment analysis (GSEA). In wild type, with log-phase cells as controls, the expression of certain COGs shifted in many comparisons (Fig. 2C): (i) protein translation (“J”) genes were repressed, with biofilms exhibiting the most downregulation, consistent with decreased protein synthesis upon nutrient depletion. (ii) Protein turnover and chaperone functions (“O”) were induced for all 24 hour cultures, indicating the stressed state of cells. (iii) All comparisons showed induction of carbohydrate metabolism and transport (“G”) genes, consistent with activation of alternative carbon source utilization pathways. (iv) Compared to log phase, genes of unknown function (“S”) also showed induction in other conditions. These results illustrate the major differences between the nutrient-depleted conditions and log-phase cells.

Other COGs changed in only specific comparisons. In stationary, genes related to “cell motility” (“N”) were induced, which in *H. influenzae* includes competence genes known to be induced in stationary phase, as well as several outer membrane autotransporter adhesins (52–54). Biofilms repressed genes involved in lipid transport and metabolism (“I”) and coenzyme metabolism (“H”), whereas genes for inorganic ion metabolism (“P”) were induced. These changes were not seen in the free-floating supernatants from the same cultures, indicating some differences between the two fractions not (Fig. 2). Full pairwise comparisons among conditions for each genotype are shown in Fig. S3, as well as comparisons of anaerobic and CDM cultures to log-phase shown in Fig. S4. Collectively, these results illustrate the large-scale gene expression differences among conditions, and they indicate some differences between well-associated and supernatant fractions.

### Genotype-dependent transcriptional differences within and among culture conditions

We next identified DEGs between Δ*msf* and wild type in each condition (Table 4; full pairwise results in Table S5). The most dramatic changes were seen in the biofilms, indicating an important role for *msf* in starved surface-attached cells. We also sought to identify DEGs between Δ*msf* and wild type that were consistent across conditions using likelihood ratio tests (LRTs). We performed LRTs with all four conditions, the two shaking conditions only, or the two static conditions only (Table 4). For all four conditions, nearly a third of genes were excluded from testing by DESeq2 as invalid due to dramatic differences between separating shaking and static culture samples (PC1 in Fig. 2A); thus, we focused on shared changes seen within the two shaking and two static culture conditions on their own. In shaking cultures, 81 genes were consistently differentially expressed, and in static cultures, there were 396 consistently differentially expressed genes. Each set is examined below, followed by an analysis of the changes in biofilms.

**TABLE 4:** Differentially expressed genes in Δ*msf* compared to wild type^*a*^

Comparison	DEGs	Up >2-fold	Down >2-fold	NAs
Exponential	313	77	47	124
Stationary	506	35	18	5
Supernatant	48	2	45	239
Well-associated	341	148	133	139
LRT all (4)	90	0	3	649
LRT shaking (2)	81	1	4	231
LRT static (2)	396	65	119	202

^*a*^ For first four rows, DEGs were determined as in Table 3 using Wald tests to identify DEGs between Δ*msf* and wild type for each individual condition. For the last three rows, LRTs were carried out to identify genotype-dependent changes consistent across conditions, using full model “~ genotype + condition” and reduced model “~ condition.” DEGs indicate the total count of differentially expressed genes with an FDR-adjusted *P*-value <0.05; Up > twofold indicates those with log_2_(FC) > 1; down > twofold indicates DEGs with log_2_(FC) < −1, and NAs indicate the number of genes with too few counts across or including extreme outliers that ruled out a meaningful statistical test.

### Differentially expressed COGs in shaking cultures

**Fig 3:**
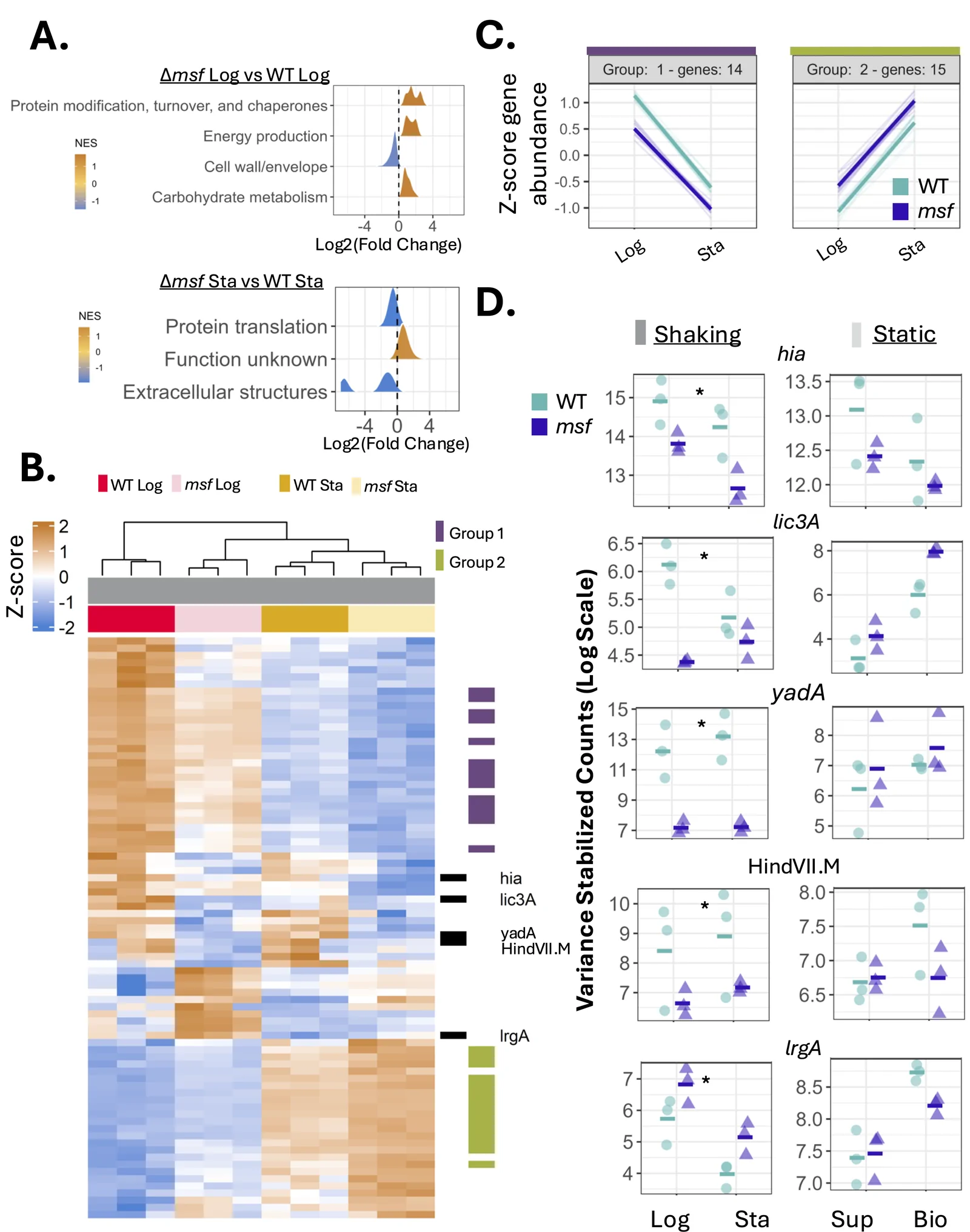
The effect of *msf* on gene expression in shaking cultures. (**A**) COG categories identified by GSEA between mutant and wild type for each condition. (**B**) Heatmap of Z-scored VST-normalized counts for the 81 significant DEGs between Δ*msf* and wild-type cells grown in both shaking cultures, determined by LRT. The black sidebar indicates the five DEGs which also had |log_2_(FC)| >1. Purple and green sidebars indicate group 1 or group 2 clusters (as in panel **C**). (**C**) Z-scored VST-normalized expression of the 29 genes clustered into two clusters of genes with similar expression patterns (purple and green sidebars in panel **B**). Color represents genotype: light blue is wild type and dark blue is Δ*ms*f. (**D**) Plot of relative expression for the five DEGs with |log_2_(FC)| >1. The left column shows relative expression in shaking cultures, and the right in static cultures; colors are the same as in panel **C**. Asterisks indicate significant DEGs as determined by LRT and adjusted *P*-value <0.05.

The effect of genotype on gene expression was evaluated within each shaking condition using GSEA of COGs (Fig. 3A; full results with “leading edge” genes for each significant category in Table S7). In exponential phase, the mutant induced three COGs consistent with increased metabolism in Δ*msf*: energy production (24 of 107 category “C” contributed to the enrichment), protein turnover (22 of 110 “O” genes), and carbohydrate metabolism and transport (31 of 113 “G” genes). Mutants also decreased expression of cell wall and envelope biogenesis genes, including many that traffic envelope-associated proteins or modulate lipooligosaccharide biosynthesis (59 of 153 “M” genes). In stationary phase, Δ*msf* induced expression of genes with unknown functions (81 of 216 “S” genes) and repressed genes involved in protein translation (98 of 211 “J” genes) and extracellular structures (3 of 7 “W” genes). These results illustrate large condition- and genotype-dependent changes in expression and highlight effects on the expression of genes associated with the cell envelope.

### Loss of *msf* consistently affects few genes in both shaking culture conditions

Although many DEGs distinguished Δ*msf* from wild type within conditions, fewer changed consistently in both exponential and stationary phases (81 genes, Table 4, row 6). These DEGs reflect genotype*-*dependent differences for cells grown in oxygenated shaking conditions despite the major difference in growth state. A heatmap of these genes is shown in Fig. 3B with details in Table S8. Clustering via DEGPatterns identified two highly co-expressed groups of genes (Fig. 3C and sidebars of Fig. 3B). Expression of genes in Group 1 was repressed in wild type between exponential and stationary and further repressed in ∆*msf* (purple, *n* = 14 genes). Expression of genes in Group 2 in wild type were induced in stationary and further induced in ∆*msf* (green, *n* = 15). Both groups included genes encoding ABC transporters and envelope-associated proteins (Table S8).

Only 5 of 81 DEGs had >2-fold changes across both exponential and stationary; one upregulated and four downregulated (Table 4, black sidebar in Fig. 3B and D). The one induced gene in Δ*msf* encodes a predicted CidA/LrgA murein hydrolase regulator involved in cell wall turnover. The four repressed genes are implicated in virulence. The two most strongly repressed encode outer membrane autotransporter adhesins: (i) Hia, a well-characterized adhesin which binds to sialylated receptors in the respiratory tract (55); and (ii) a paralogous autotransporter related to YadA, whose homolog in *Yersinia* is required for gut colonization (56, 57). These findings suggest that *msf* influences the expression of autotransporter adhesins. Notably, *hap* was not significantly affected despite the putative Y2H interaction, though other autotransporters were differentially expressed (Fig. S5), indicating specific variable effects of *msf* on the expression of this gene family.

The other two repressed genes were *lic3A,* encoding a lipooligosaccharide sialyltransferase linked to complement resistance (58, 59), and a DNA methyltransferase (60–62). Because both these loci are phase variable (59, 63), RNA-seq reads across their repeat tracts were examined, with no strain differences detected (Fig. S6). Together, these results show that *msf* affects gene expression in planktonic cultures, particularly membrane and envelope-associated genes. A small subset of genes which encode autotransporter adhesins was strongly affected in both log- and stationary-phase but not consistently altered in the 24 hour cultures (Fig. 3D).

### Loss of *msf* has large impacts on gene expression in biofilm cells

**Fig 4:**
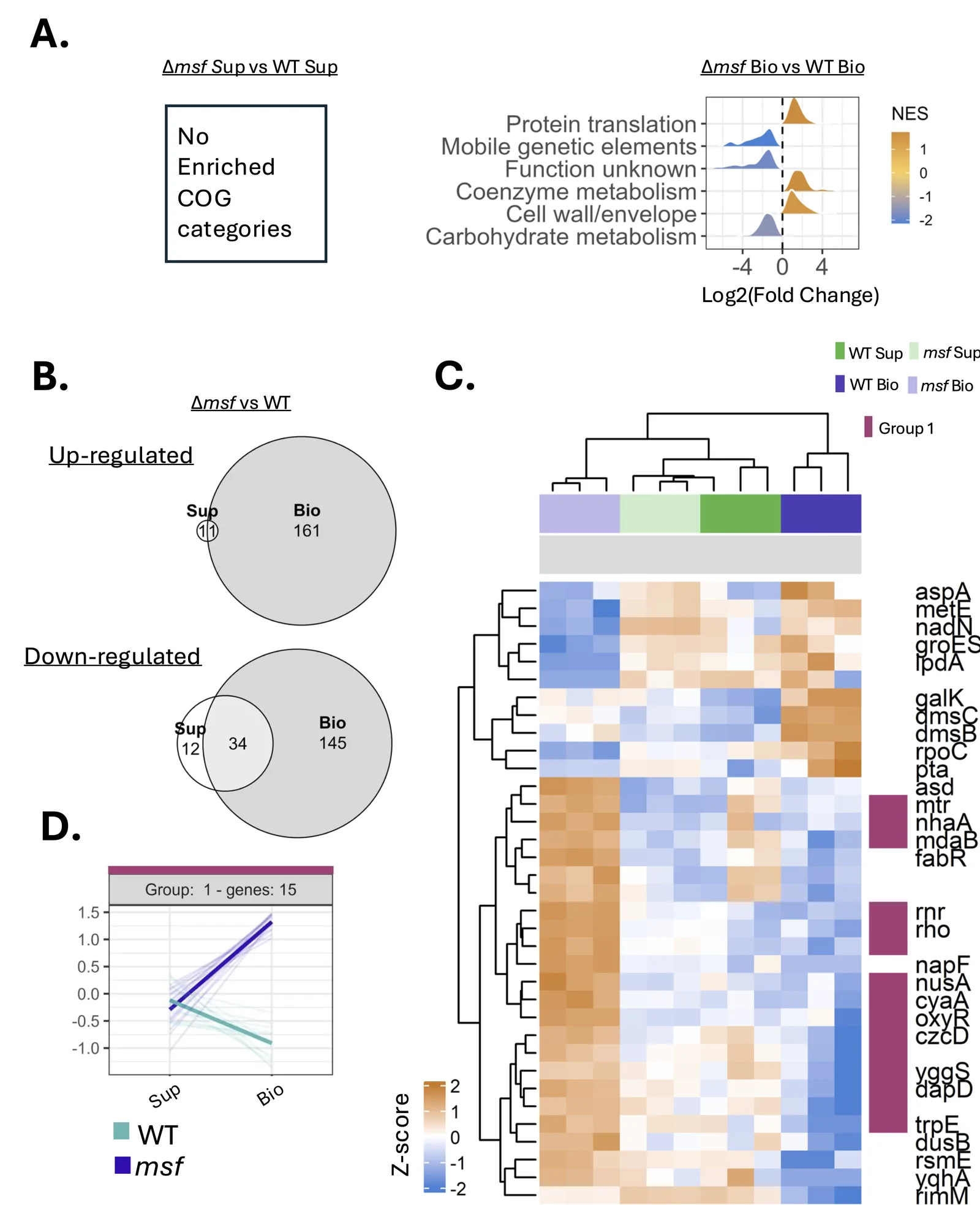
The effect of *msf* on gene expression in well-associated biofilm cells. (**A**) Ridge plots of enriched COG categories between mutant and wild-type biofilm fractions determined by GSEA. (**B**) Venn diagram of DEGs from the pairwise comparisons of mutant versus wild type in both supernatant and well-associated biofilm fractions. (**C**) Heatmap of Z-scored VST-normalized gene expression for 35 DEGs identified as significantly differentially expressed due to an interaction in the mutant well-associated biofilm condition. Upregulation is indicated with orange and downregulation with blue. Columns represent samples and rows represent genes. Vertical sidebar in magenta indicates genes as clustered in panel **D** (*n* = 15). (**D**) Z-scored VST-normalized expression of 15 genes with similar patterns of expression in two static fractions. The heavier lines represent the average of all genes, with light blue representing the WT and dark blue representing *msf*. These 15 genes are highlighted in panel **C** with the magenta sidebar.

We next examined the effects of Δ*msf* in the two static culture conditions. As noted above, Δ*msf* had a stronger effect on well-associated biofilm than free-floating supernatant cells. Wild type showed similar expression patterns in both fractions, whereas the mutant displayed major changes in the biofilm, with far fewer differences in the supernatant (Fig. 2; Tables 3 and 4). Although 396 DEGs were found by LRT, most of the changes were due to changes in the biofilms (Fig. S5; Table S9). These results indicate an especially important role for *msf* in surface-attached biofilm cells. Consistent with this observation, GSEA identified several enriched COG categories in the biofilms but none in supernatants (Fig. 4A; Table S6). Compared to wild type, Δ*msf* biofilms downregulated mobile genetic elements (28 of 44 “X” genes), genes of unknown function (71 of 213 “S”), and carbohydrate metabolism and transport genes (38 of 113 “G”). Upregulated COGs included protein translation genes (96 of 211 “J”), coenzyme metabolism genes (38 of 98 “H,” notably genes involved in thiamine biosynthesis), and cell wall/envelope biogenesis genes (63 of 153 “M,” notably lipid A biosynthesis genes). Many enriched genes were predicted to be envelope-localized or involved in membrane assembly.

Further illustrating the impact of Δ*msf* on biofilms, the Venn diagram in Fig. 4B shows that while some DEGs overlapped between fractions, most changes occurred in well-associated cells (Table S10). Therefore, we next tested for genes whose expression depends on an interaction between genotype and condition.

### Loss of *msf* causes well-associated biofilms to differentiate from free-floating supernatants

To assess how *msf* alters gene expression between the two static conditions, an LRT identified 35 genes with significant genotype-by-environment interactions. The heatmap in Fig. 4C shows strong opposing genotype effects in biofilm cells but similar expression in supernatants. The 35 genes were not enriched for a specific COG, though several were involved in transcription and energy production and conversion. Fifteen genes formed a co-expressed cluster (Fig. 4D; magenta-colored sidebar on Fig. 4C), for which wild type was strongly repressed in well-associated biofilms relative to supernatants, while Δmsf cells strongly induced them. This cluster included genes involved in transcription (*rnr*, *oxyR*, *rho*, *nusA*) and energy-related genes (*nhaA*, *dmsB*, *dmsC*, *napF*). Predicted functions for all 35 are in Table S11. These genes indicate that *msf* may play a role in “buffering” differences in surface-attached compared to free-floating starved cells.

### Partially overlapping differential expression in a knock-in mutant of the lab strain Rd

To test whether *msf* has similar effects in a different genetic background, we used RNA-seq to compare strain Rd KW20, which lacks a distributed SLR-containing gene, and a derivative carrying one copy of *msfA1* inserted at *ompP1* (32). Triplicate samples were collected and analyzed from mid-log phase and well-associated biofilms. DEGs between *msf*-negative (wild-type Rd) and *msf-*positive (*ompP1::msfA1*) were identified (Table S12). To compare results between backgrounds, orthologs between Rd and PittII were determined using Panaroo (Table S13). The Venn diagrams in Fig. S7 show a partial overlap of DEGs between strains, with intersecting genes changing in the same direction. However, most DEGs were strain-specific, suggesting the effects of *msf* depend on both environment and genetic background. Several caveats apply: *msf* insertion also disrupts *ompP1*, a bifunctional adhesin/fatty acid transporter (64); only one copy of *msf* was added to Rd versus four copies deleted from PittII; protocols slightly differed between experiments (rRNA depletion method); and cross-strain transcriptome comparisons are complicated by differences in gene content.

### Little or no impact of *msf* on viability, biomass, or metabolic activity in static cultures

**Fig 5:**
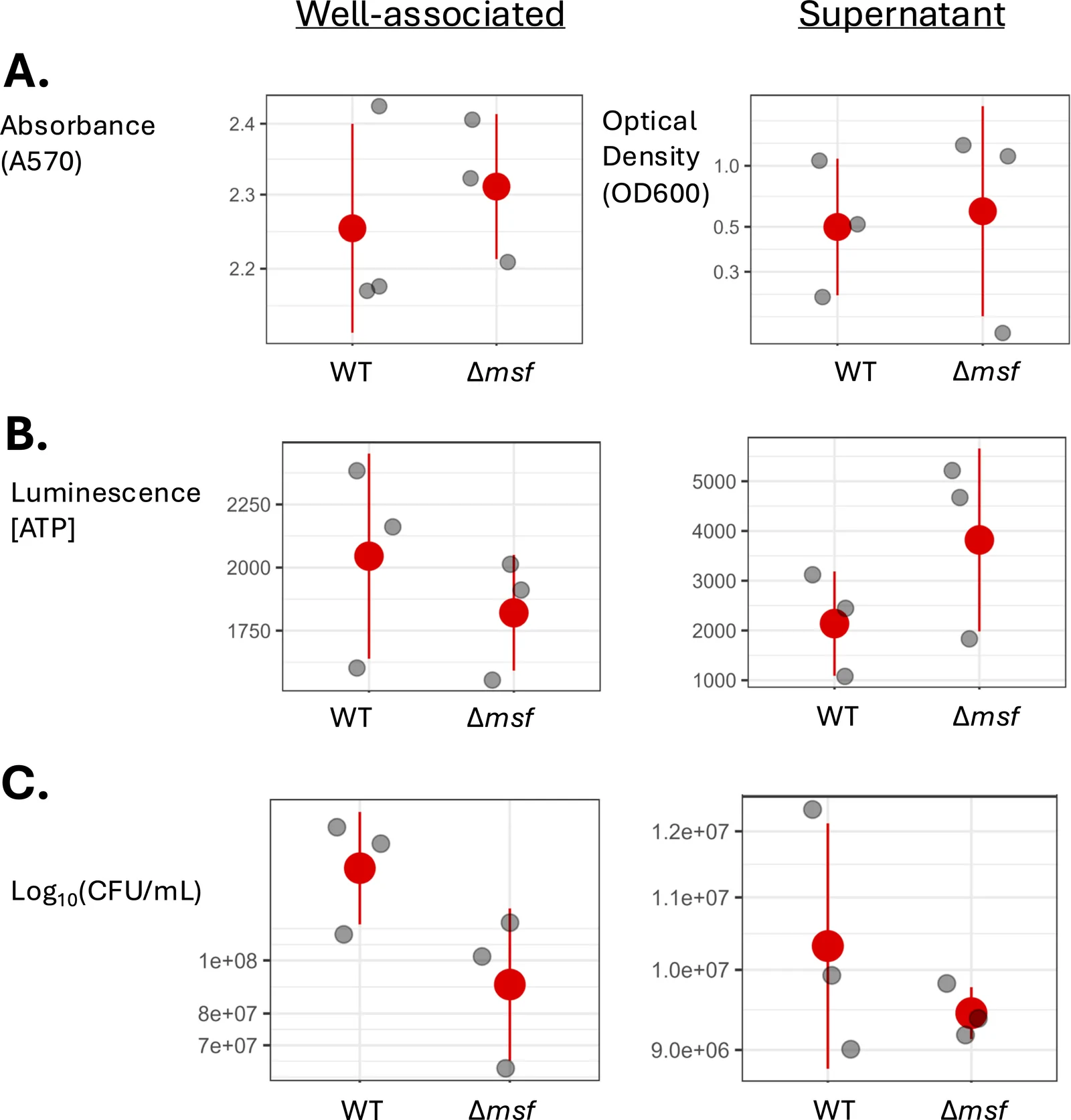
Phenotypic assays of 24 hour static cultures with or without *msf*. (**A**) Crystal violet staining of wild-type and mutant 24 hour static culture biofilms and OD_600_ measurements of 24 hour supernatant fractions. (**B**) Luminescence proportional to the concentration of ATP in the cultures. (**C**) Colony-forming units (CFU) per milliliter of well-associated biofilm and supernatant cultures grown in static conditions for 24 hours. One-way ANOVA was used to determine significant differences between genotypes for each assay and fraction; CFU per milliliter measurements were log-transformed before statistical testing.

To determine whether the transcriptional differences seen by RNA-seq resulted in phenotypic changes in the 24 hour static cultures, we compared Δ*msf* and wild-type PittII cells using several assays (Fig. 5). Measurements of biofilm biomass by crystal violet staining (CV *A*_570_) and supernatant biomass by optical density (OD_600_) showed no significant differences. The concentration of ATP measured using a luminescence assay also showed no differences, although a trend toward increased [ATP] in the supernatant was seen (one-way ANOVA *P* = 0.069). Lastly, CFU in each fraction were measured, again showing no significant changes. We also assayed Rd KW20 and its *msf* knock-in mutant derivative for these phenotypes, along with a Δ*ompP1* mutant as a control for the effects of inserting *msf* at this locus. This showed no significant effects of *msf,* except a slight decrease in crystal violet biofilm staining due to the knock-in independent of *ompP1* loss-of-function (Tukey’s HSD *P*-value = 0.020; Fig. S8); other significant differences from wild type were also seen in Δ*ompP1*. These results indicate that *msf* has little or no impact on biofilm biomass, metabolic activity, or viable cell density, though many other biofilm phenotypes have not been tested.

## DISCUSSION

Even as genome sequencing efforts have enabled association studies to identify new virulence genes, large numbers of conserved bacterial genes have under- or unannotated functions. Here, we used orthogonal naïve approaches to shed light on the function of *Haemophilus influenzae* SLR-containing genes, previously associated with clinical disease and intracellular survival. Little else has been known other than their possession of a signal peptide and Sel1-like repeats. Here, we report identification of putative physical interactions of Msf with envelope-associated proteins, as well as transcriptome-level differences that depended on culture condition and especially impactful in surface-attached biofilm cells. Together, the results indicate a role for Msf at the cell envelope, possibly participating in membrane protein trafficking within the periplasm. However, our findings raise more questions than they answer.

We found evidence for protein-protein interactions using a commercial Y2H screen of Msf with LolD (the cytoplasmic homodimeric ATP-binding cassette of the lipoprotein trafficking ABC transporter LolCDE) and Hap (an OM, monomeric autotransporter adhesin). Validation of these interactions will require independent experiments, and specific experiments are also needed to confirm how Msf is trafficked and localized in cells. Our results collectively support a role for Msf at or beyond the cell envelope; however, LolD and Hap have distinct localizations and functions that, together with the RNA-seq results, suggest at least three possible models for its function.

Lipoproteins destined for the OM are processed by LolCDE, which extracts triacylated lipoproteins from the periplasmic face of the IM and transfers them to the periplasmic LolA chaperone protein (41, 43, 65–68 ). An interaction with the LolD ATPase could indicate that Msf functions to modulate lipoprotein trafficking. However, this model is at odds with LolD’s localization at the inner face of the IM and Msf’s N-terminal SP1 signal peptide, which predicts processing by Sec. Thus, although in principle Msf may have transient interactions with LolD prior to trafficking into the periplasm, the Msf-LolD interaction may instead be an artifact of the Y2H system.

The Hap autotransporter adhesin is a well-characterized OM protein with an SP1 signal peptide like Msf, where it promotes bacterial autoaggregation and adherence to airway cells (47, 48, 67, 68). Following Sec-dependent trafficking through the IM, Hap is assembled into the OM by the BAM complex, a periplasmic chaperone responsible for transport and assembly of beta-barrels (69–77). Mature Hap is embedded in the OM via its beta-barrel, and the N-terminal passenger domain with protease and adhesin activities projects into the extracellular space. Y2H supported a specific interaction of Msf with Hap at the coiled-coil immediately N-terminal of the autotransporter and overlapping the autoproteolytic cleavage site. We think the most likely function for Msf is as a periplasmic chaperone-like protein that modulates the export of OM proteins, consistent with a periplasmic interaction between Msf and Hap. Alternatively, Msf may associate with Hap outside the cell to modulate its autoproteolytic activity. Either function could have strong effects on global gene expression, including other autotransporter adhesins, by changing surface protein expression or regulating cell-to-cell contacts.

The transcriptional changes observed for many envelope-associated genes, including for several autotransporter adhesins, support a role for Msf in processing surface-associated proteins. Such a role for Msf is consistent with the condition-specific effects we observed by RNA-seq, particularly differential expression of genes involved in the cell envelope, membrane function, and cell wall biogenesis. The most pronounced effects were seen in the 24 hour static conditions, where loss of *msf* led to strong divergence between surface-associated biofilm and the free-floating supernatant. Notably, Δ*msf* biofilms upregulated genes repressed in the wild type, including *oxyR* and several periplasmic and membrane-associated redox proteins. In *Neisseria meningitidis*, knockout of the SLR protein NMB0419 causes widespread transcriptional changes, particularly in the Fur regulon (78, 79). Importantly, NMB0419’s activity depends on its signal peptide, supporting a periplasmic or extracellular role with large indirect effects on transcription (33, 69, 78). Profiling the surface proteome of cells with or without *H. influenzae msf* would clarify our hypothesized function.

While this study focused on *msf,* a specific SLR gene found in only some strains, a core SLR-containing gene is conserved across *H. influenzae* (32). The function of this core SLR-containing gene remains undefined, and its essentiality is unclear from existing Tn-seq studies (70, 80). Furthermore, SLR genes in *H. influenzae* have substantial allelic variation in copy number, repeat number, and amino acid sequence, suggesting that these genes may not all be functionally equivalent.

In summary, our results support an important role for SLR-containing genes in *H. influenzae* in modulating activities at the cell envelope, and we propose that Msf may be a periplasmic protein that functions in OM protein trafficking. Although we did not observe any dramatic changes in biofilm viability or biomass, the large-scale transcriptional changes suggest that further phenotypic assays of biofilms are warranted. Further defining the roles of virulence-associated distributed genes like *msf* could provide new insights into poorly characterized protein functions, offer potential new molecular diagnostics of pathogenic potential, or suggest new routes for therapeutic interventions.

## MATERIALS AND METHODS

### Bacterial strains and culture

*Haemophilus influenzae* PittII (BS434, or WT for wild type) was originally isolated from a child with otorrhea and encodes four tandem copies of *msf* of the SlrVA subfamily (2, 5, 28). The mutant (GC598, or Δ*msf*) had all four tandem copies of *msf* deleted using a spectinomycin resistance cassette (Spc^R^) (28, 32). The Rd KW20 laboratory strain and a single-copy knock-in were also used (GC594) (17). The single-copy knock-in was into *ompP1*, so additionally Δ*ompP1::CmR* (GC1052) was used. Bacteria were cultured at 37°C in brain heart infusion broth supplemented with 10 µg/mL hemin and 2 µg/mL β-nicotinamide adenine dinucleotide (sBHI) or in CDM (71, 72).

### Yeast two-hybrid protein-protein interaction screen

An *H. influenzae msf1A* clone from PittII (pgaptmp_1629 in Table S4) (17) was submitted to Hybrigenics, Inc. for the ULTImate Y2H SCREEN (39, 40) to identify protein-protein interactions. The MsfA1 bait was fused to a LexA DNA-binding domain, while prey libraries were expressed as fusions to a Gal4 transcriptional activation domain. Interaction between bait and prey activates transcription of a *HIS4* reporter. Hybrigenics, Inc. screened a prey library derived from THP-1 cells, then with a custom prey library generated from genomic DNA of 11 phylogenetically diverse *H. influenzae* isolates. Positive interactions were identified by growth on selective media (His^+^, Leu^+^, Trp^+^). Hybrigenics performed Sanger sequencing from both ends of the prey inserts to infer full-length prey fragment sequences of the 360 clones (Table S1).

To identify putative interactors, unique prey sequences were mapped using BLASTn against a database of clustered protein-coding genes from *H. influenzae* (81). Fragments were filtered to retain those that were in-frame, spanned a single gene (100% query coverage), and had 100% identity. Interaction confidence was further assessed using the “predicted biological score” (PBS), which evaluates the likelihood of “true” interactions based on recurrent independent prey mapping to the same gene (39, 40). Each prey’s PBS scores range from A (high confidence) to D for (low confidence), and F indicates known artifacts (i.e., reporter activation by LexA prey). Annotation of protein domains used InterPro (74, 82). Overlapping prey fragments mapping to a single gene were intersected to define the minimal shared interval on the prey protein that interacts with Msf.

### Conditions for RNA-seq transcriptome profiling

Cells were harvested for RNA-seq from triplicate WT and Δ*msf* cultures (83, 84). First, for shaking cultures, overnight cultures were diluted 1:50 and incubated in sBHI or CDM with shaking at 250 RPM until mid-log (OD_600_ = 0.3, ~2.5 hours) or stationary (OD_600_ = ~0.8–1.0, ~6 hours). Anaerobic cultures were grown in sBHI using Forma Scientific, Inc. Model 1025 anaerobic chamber and 1 mL was harvested 2.5 and 6 hours after subculture, representing early- and late-logarithmic phase. For biofilms, overnights were diluted 1:50 into 1 mL sBHI in 48-well plates, then incubated without shaking (static) under 5% CO_2_ for 24 hours. The 24-hour static cultures were divided into two fractions: free-floating cells and aggregates (the “supernatant”) and the well-associated cells (the “biofilm”) after gentle washing in 1×PBS, 0.1×BHI. In total, WT and Δ*msf* were tested in eight conditions: log-sBHI, stationary-sBHI, log-CDM, stationary-CDM, early-anaerobic sBHI, late-anaerobic sBHI, 24 hour sBHI static culture supernatant, and 24 hour sBHI well-associated biofilm (Table 1). A smaller experiment used Rd KW20, which lacks *msf*, and knock-in GC594, and collected log-sBHI and 24 hour sBHI well-associated biofilm samples in triplicate.

### RNA extractions, libraries construction, and sequencing

Pelleted cells were resuspended in 200 µL RNAprotect (Qiagen) to lyse cells and stabilize RNA before extraction using a Qiagen RNeasy RNA extraction kit. Quantity and quality were evaluated using NanoDrop spectrophotometry, Qubit fluorometry, and Agilent 2100 Bioanalyzer. Sequencing libraries from PittII and its Δ*msfA1-4::specR* knockout derivative (BS434 and GC598, respectively) used Illumina stranded mRNA library kits with RiboZero (epidemiology) depletion to remove highly abundant rRNAs prior to cDNA synthesis. The poly-A selection step was omitted. Libraries were sequenced at 1 × 75 nucleotide single-end reads using Illumina NextSeq 500 with a target coverage of ~1 million reads per library. The experiment with Rd and the GC594 knock-in derivative was processed similarly, except instead of RiboZero depletion, JumpCode Genomics CRISPRclean (epidemiology) kit was used after cDNA library construction and pooling.

### PittII reference genome

We produced a new sequence for PittII using Pacific Biosciences Sequel sequencing using manufacturer’s recommendations to produce a high-quality finished genome assembly. The genome was annotated using the Prokaryotic Genome Assembly Pipeline (PGAP) from NCBI (75).

### RNA-seq read processing

Sequence reads were aligned to either PittII (DDBL/ENA/GenBank accession JBYGJL010000000) or Rd KW20 (GenBank: GCA_000027305.1, recircularized to *dnaA* and replacing non-ACGTN characters with Ns) reference assemblies using BWA with default settings; SAMtools (v.1.21) was used to compress, sort, and evaluate the resulting alignments (85–87. To assemble a count table of RNA-seq reads aligned to each gene in each sample, bedtools coverage was used with Prokka annotations for all gene and pseudogene features (88, 89). A summary of sequencing metrics is in Table S3. The quality of rRNA depletions was evaluated using read counts at the 19 rRNA genes, then these genes were excluded prior to downstream differential expression analysis. For PittII, the seven ORFs in the *msf* operon were excluded from the final count table, since the mutants all lacked them. The final set of 1,960 gene features was included in the count table used for differential expression and pathway analysis (Table S4). For the Rd experiment, the count table is in Table S12.

### Differential expression analysis

Statistical analyses of RNA transcript abundances were performed using R (v.4.0.0) and RStudio (v.4.2.1). Differential expression was determined using DESeq2 with shrinkage of variance performed using ApeLGM (49, 90, 91). Pairwise comparisons used Wald tests, and LRTs were performed to identify genes showing (i) genotype-dependent chafanges across environments, and (ii) genotype-by-environment interactions. The Benjamini-Hochberg procedure was used to decrease the false discovery rate, and adjusted *P*-values of <0.05 were deemed significant. Genes found to have significant genotype-by-environment interactions were clustered into sets with similar expression patterns using the DEGpatterns function in DEGreport (92).

### Gene set enrichment analysis for COG categories

For annotation of protein-coding genes, eggNOG mapper tool (v.2.1.12) was used with “sensitive” settings (50) to identify COG IDs, then define COG categories (51). Differential expression of COGs used ClusterProfiler to perform GSEA or overrepresentation analysis (ORA) (93–97). Genes with no COG assigned by eggNOG were given COG category “S,” which represents “Function unknown.” Genes that DESeq2 gave adjusted *P*-values of NA were excluded from GSEA, since these had unreliable log_2_(fold change) due to low expression across samples or had extreme outliers (49). For ORA, subsets of genes were grouped—either because up- or downregulated (*P*.adjusted <0.05) or identified as co-expressed by DEGpatterns. For a given group, ClusterProfiler’s enricher function was used to calculate whether specific COG categories were enriched compared to the background list of all genes.

### Transcriptome comparisons between strain backgrounds

A pangenome was made using the PGAP-annotated genomes of PittII and Rd KW20 using the Panaroo pipeline (88, 98) using default parameters (Table S13). If available, the gene cluster identified via Panaroo was used to compare the DEG lists of each strain’s transcriptome. The “control” in the Wald test was with *msf* present: PittII WT or GC594 (Rd *msf* knock-in), and the “treatment” was the absence of *msf:* GC598 (PittII *msf* knockout) or Rd WT.

### Phenotypic assays for colony-forming units, biomass, and metabolic activity

Overnights of wild type and mutant were diluted 1:50 in sBHI in 200 µL into wells of a 96-well plate and grown at 37°C for 24 hours without shaking under 5% CO_2_ before processing.

To estimate viable cell density, CFU per milliliter were determined. Supernatants were gently transferred off the biofilm to 1.5 mL microcentrifuge tubes, pelleted, and resuspended in dilution solution (9:1 PBS:sBHI). The wells were gently washed with dilution solution to remove unattached cells. Next, dilution buffer was added with DNase I (125 µg/mL) to resuspend the biofilm cells for 1 hour at 37°C with 5% CO_2_. At 30 and 60 minutes, wells were scraped with a pipette tip. A serial dilution and drip titer were performed on supernatant and biofilm fractions, and sBHI plates were incubated for 24 hours at 37°C under 5% CO_2_. The next day, colonies were counted and the CFU per milliliter was calculated as total counted colonies divided by the total effective volume plated.

For biomass measurements, crystal violet staining was performed. The supernatant was separated from the well-associated biofilm and transferred to a new 96-well plate. The OD_600_ of the supernatant was measured as an estimate of overall supernatant biomass. The biofilms were gently washed with sterilized Milli-Q water (Sigma-Aldrich Millipore Advantage A10) then treated with 0.05% crystal violet for 20 minutes. After treatment, biofilms were washed three times with water. To solubilize biofilms, each well in the 96-well plates were incubated with ethanol:acetone (4:1) for 20 minutes shaking at 50 RPM at room temperature. The *A*_570_ was measured to calculate biofilm biomass.

To estimate metabolic activity, a luciferase-based assay was used to measure the relative concentration of ATP. The supernatant was transferred to a white-bottomed 96-well plate, and an equal volume of BacTiter-Glo reagent (Promega) diluted 1:40 in 1× PBS was added. Biofilms were washed twice with 1× PBS, then resuspended in diluted BacTiter-Glo. Both supernatant and biofilm plates were incubated at room temperature on an orbital shaker at 100 RPM for 15 minutes. The resuspended biofilms were transferred to the white-bottomed 96-well plate. Luminescence was measured using a Tecan Infinite M200 PRO.

Assays were performed in triplicate, and differences between genotypes for each phenotype were determined by one-way ANOVA with *P*-value <0.05 deemed significant. For CFU per milliliter values, data were log transformed prior to testing.

## Data Availability

The complete assembly of PittII was deposited as DDBJ/ENA/GenBank accession JBYGJL010000000. RNA-seq data (raw reads and count tables) were deposited at the Gene Expression Omnibus (GEO) under accession number GSE330915. Table S1 contains the full prey fragment sequences for all recovered clones from the Hybrigenics Y2H screen, as well as the best BLASTn hit to clustered *H. influenzae* protein-coding genes. Table S14 and Table S12 contain BED6 columns reporting the gene coordinates and names used for the PittII and Rd KW20 experiments, along with the inferred COG categories and the full RNA-seq count table appended as additional columns, and Table S13 provides a lift-over table for orthologs between the two.

## SUPPLEMENTAL MATERIAL

Supplemental figures mbio.01263-26-s0001.pdf Fig. S1 to S9.

Supplemental captions mbio.01263-26-s0002.pdf Captions to Tables S1 to S13.

Supplemental tables mbio.01263-26-s0003.xlsx Tables S1 to S13.

ASM does not own the copyrights to Supplemental Material that may be linked to, or accessed through, an article. The authors have granted ASM a non-exclusive, world-wide license to publish the Supplemental Material files. Please contact the corresponding author directly for reuse.
