# Supplementary material for: The msf gene causes condition-specific shifts in global gene expression in Haemophilus influenzae
Source: mBio. 2026 Jul 29;17(9):e01263-26. doi: 10.1128/mbio.01263-26 (PMC13556256; doi:10.1128/mbio.01263-26)
Supplement: Supplementary file 1 [file mbio.01263-26-s0001.pdf]

Supplementary Figures

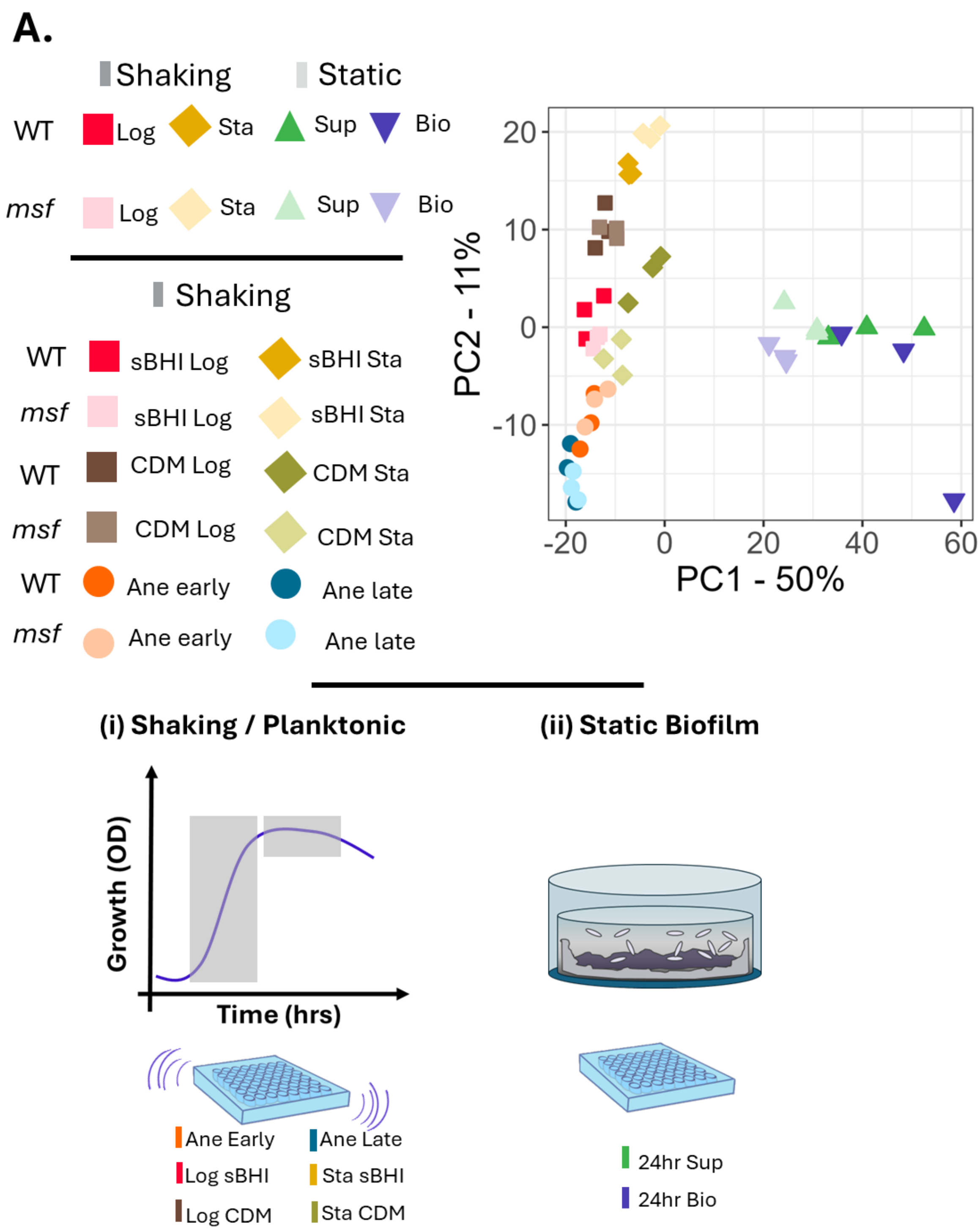

Figure S1 – PCA including all in vitro conditions. A. Principal component analysis (PCA) which contains all eight conditions, or B. just the shaking conditions examined in this experiment: exponential and stationary phase in both sBHI and CDM media, early logarithmic and late logarithmic phase in anaerobic cultures, 24-hour well-associated biofilms and 24-hour supernatant. Color and shape distinguish condition: circles are anaerobic (blue 'early', orange 'late'); squares represent exponential (red 'sBHI', brown 'CDM'); diamonds are stationary (gold 'sBHI', yellow 'CDM'); for 24-hr static cultures, green triangles indicate free-floating supernatant, purple inverted triangles indicate well-associated biofilm. Genotypes are shown with filled (wild type) or open (Δmsf) symbols.

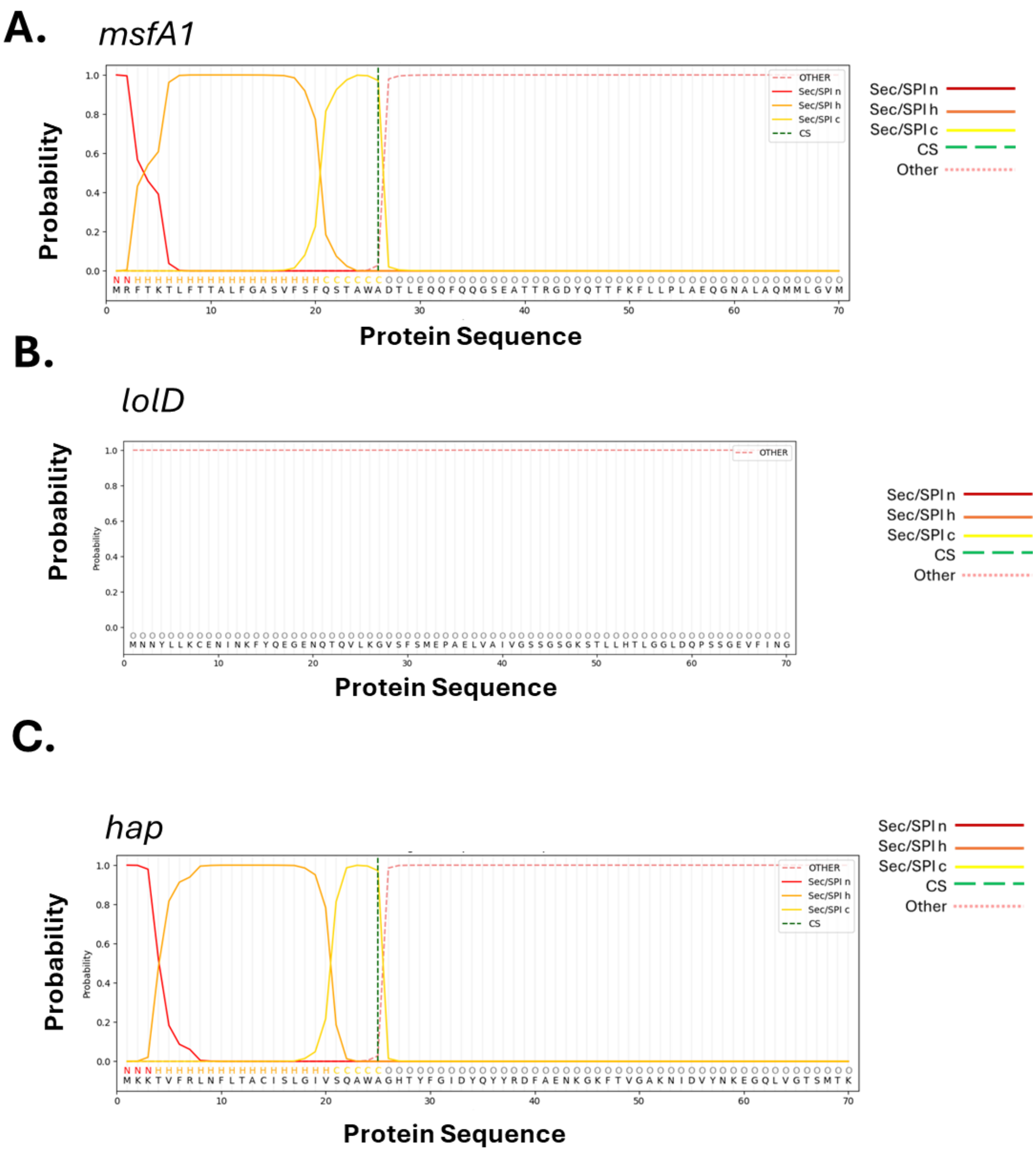

Figure S2 – Signal peptide prediction. A. Protein sequences were analyzed using SignalP v6.0 to identify N-terminal signal peptides. Predicted signal peptides are indicated, with cleavage sites annotated based on algorithm scores. A. msfA1 B. lolD C. hap.

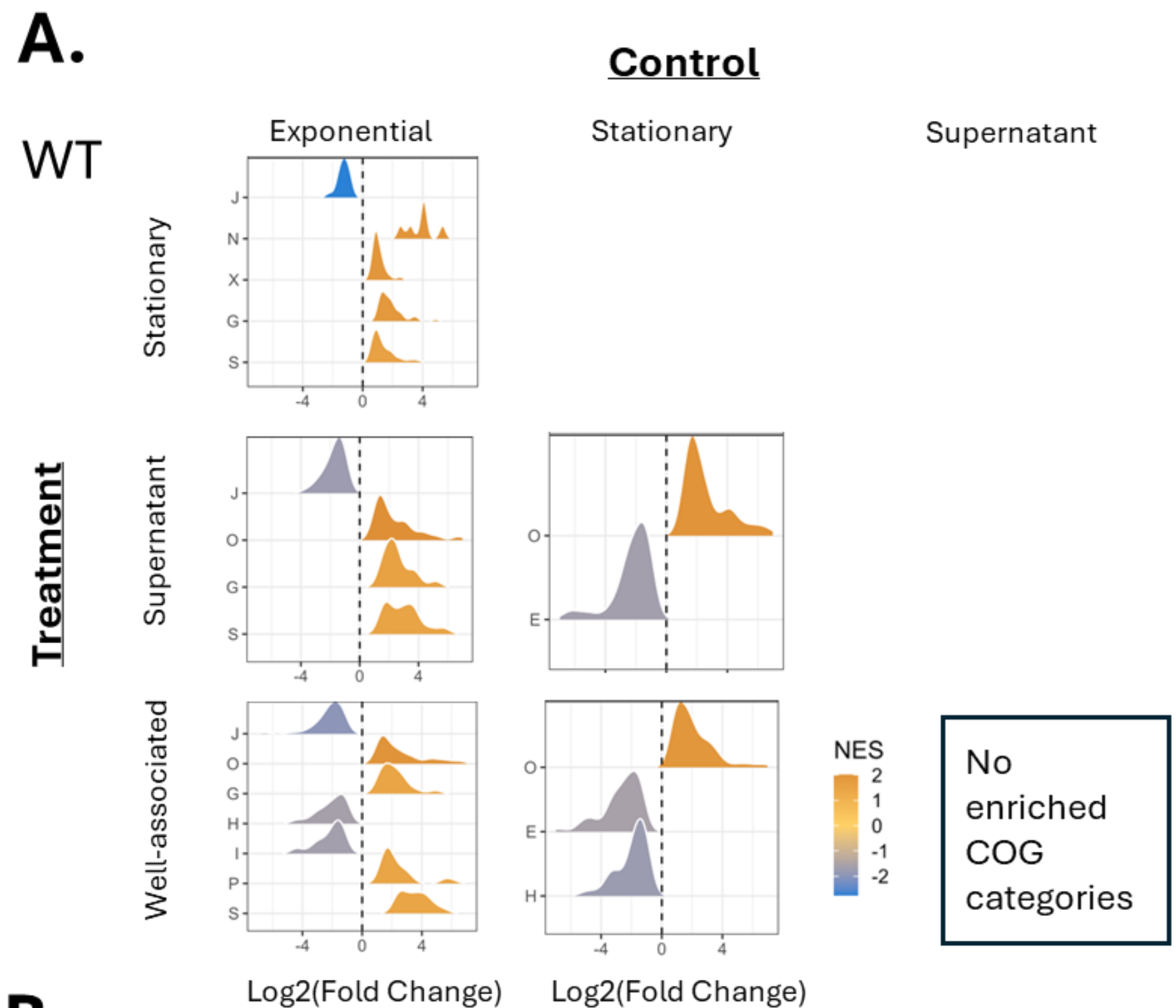

General Category	COG ID	Description
I. Information storage and processing	J	Translation, ribosomal structure, and biogenesis
	K	Transcription
	T	Signal transduction mechanisms
	M	Cell wall/membrane/envelope biogenesis
	N	Cell motility
	W	Extracellular structures
	U	Intracellular trafficking, secretion, and vesicular transport
II. Cellular processes and signaling	O	Post-translational modifications, protein turnover, chaperones
	X	Mobile genetic elements
	C	Energy production and conversion
	G	Carbohydrate transport and metabolism
	E	Amino acid transport and metabolism
	H	Coenzyme transport and metabolism
	I	Lipid transport and metabolism
III. Metabolism	P	Inorganic ion transport and metabolism
	R	General function prediction only
IV. Other	S	Function unknown

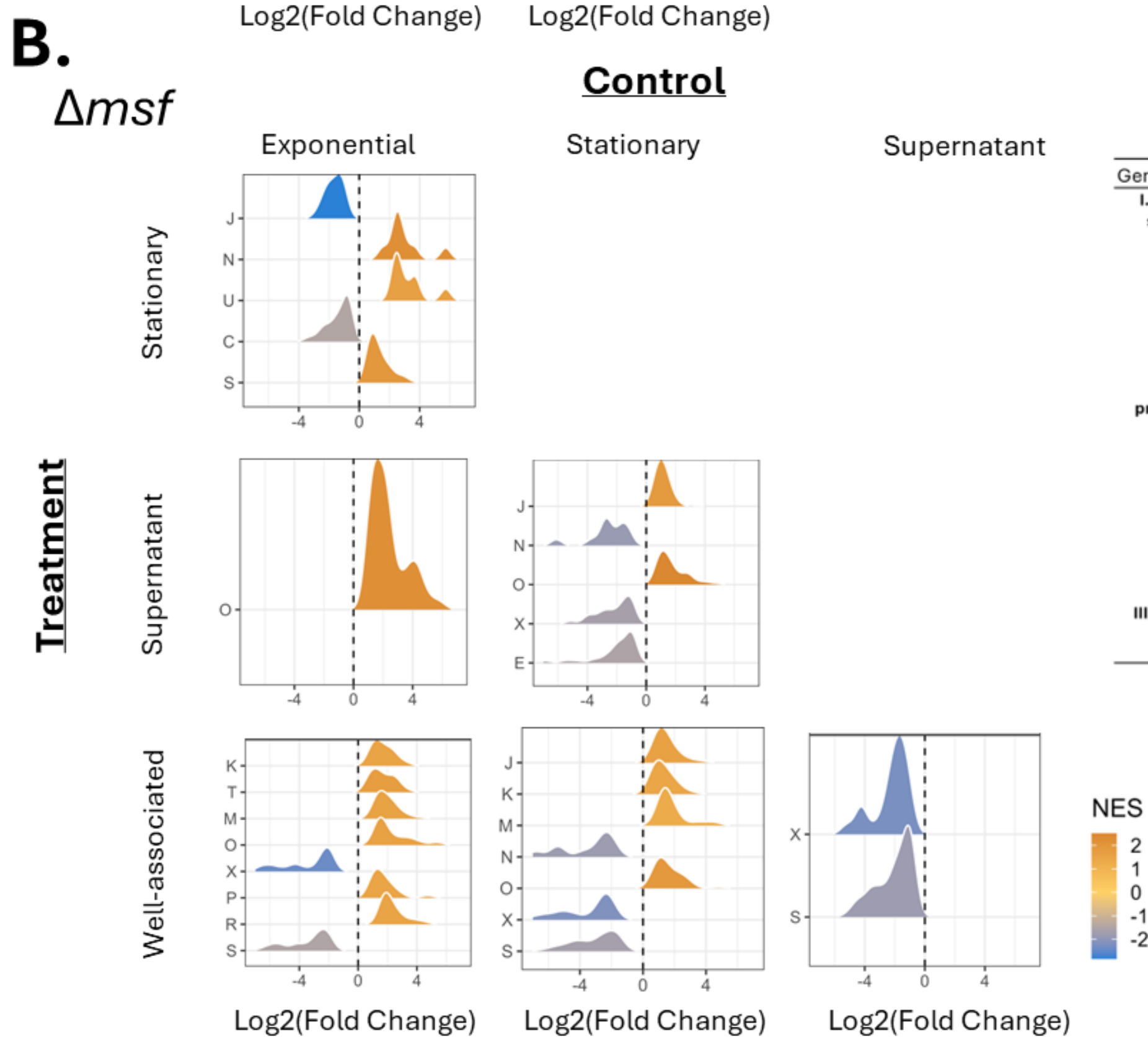

General Category	COG ID	Description
I. Information storage and processing	J	Translation, ribosomal structure, and biogenesis
	K	Transcription
	T	Signal transduction mechanisms
	M	Cell wall/membrane/envelope biogenesis
	N	Cell motility
	W	Extracellular structures
	U	Intracellular trafficking, secretion, and vesicular transport
II. Cellular processes and signaling	O	Post-translational modifications, protein turnover, chaperones
	X	Mobile genetic elements
	C	Energy production and conversion
	G	Carbohydrate transport and metabolism
	E	Amino acid transport and metabolism
	H	Coenzyme transport and metabolism
	I	Lipid transport and metabolism
III. Metabolism	P	Inorganic ion transport and metabolism
	R	General function prediction only
IV. Other	S	Function unknown

Figure S3 – Between-condition pairwise comparisons. Pairwise comparisons between A. early and late anaerobic cultures or B. exponential or stationary cultures in CDM for wild-type cells with exponential cultures in sBHI media as controls. The x-axis is the rank-ordered log2(fold-change) of the 1898 coding genes (excluding the ORFs in the msf operon). Density histograms are for the "leading edge" genes that contribute to the skewed distribution of that category. Fill color is the normalized enrichment score (NES).

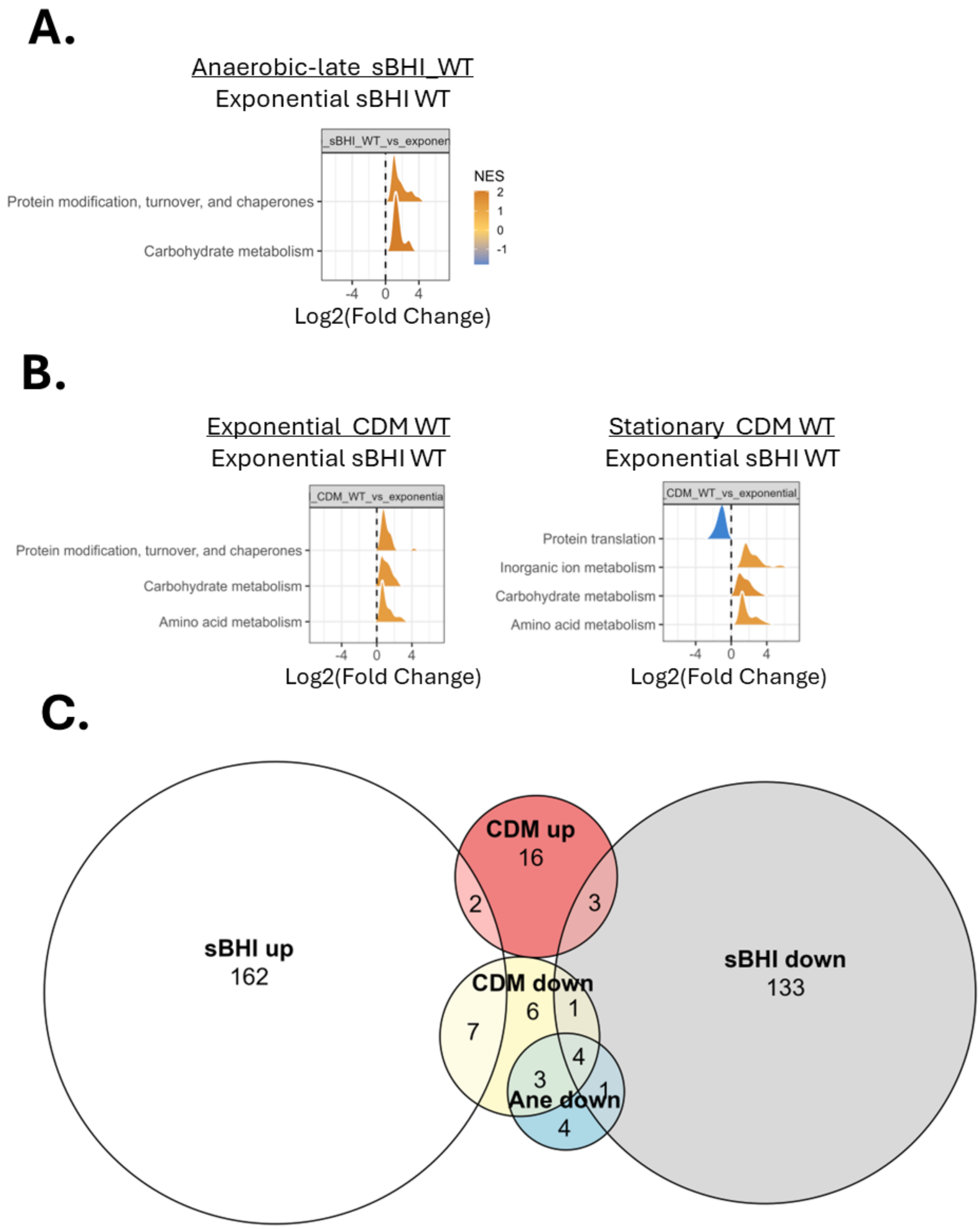

Figure S4 – Consistent changes in shaking culture conditions. Pairwise comparisons between A. early anaerobic cultures or B. exponential or stationary cultures in CDM for wild-type cells with exponential cultures in sBHI media as controls. The x-axis is the rank-ordered log2(fold-change)

of the 1898 coding genes (excluding the ORFs in the msf operon). Density histograms are for the "leading edge" genes that contribute to the skewed distribution of that category. Fill color is the normalized enrichment score (NES). C. Venn diagram of the DEGs between msf and WT in all exponential cultures (sBHI, CDM, early-anaerobic).

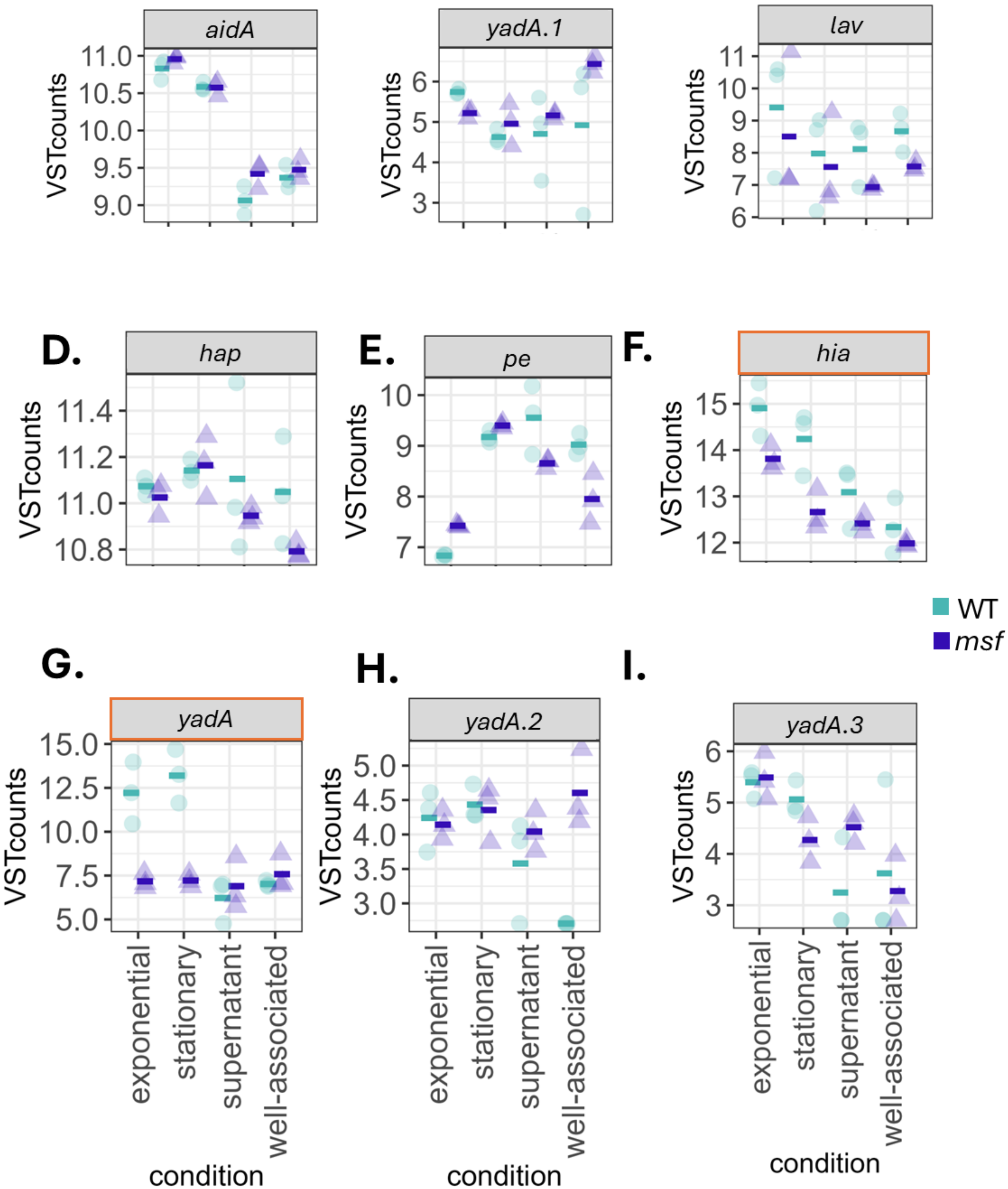
aidA
yadA.1
lav
D.
hap
E.
pe
F.
hia
WT
msf
G.
yadA
H.
yadA.2
I.
yadA.3
VSTcounts
exponential
stationary
supernatant
well-associated
condition

Figure S5 – Adhesin expression between genotypes. Variance-stabilized (VST) counts from DESeq2 are shown for all samples across the four main experimental conditions. Each point represents the normalized expression level of nine adhesins present in the PittII genome. In each plot, wildtype is represented by the green circles, and msf is represented by the purple triangles, with a bar to indicate the means of the replicates. The adhesins in the main manuscript, identified as significantly differentially expressed, are highlighted.

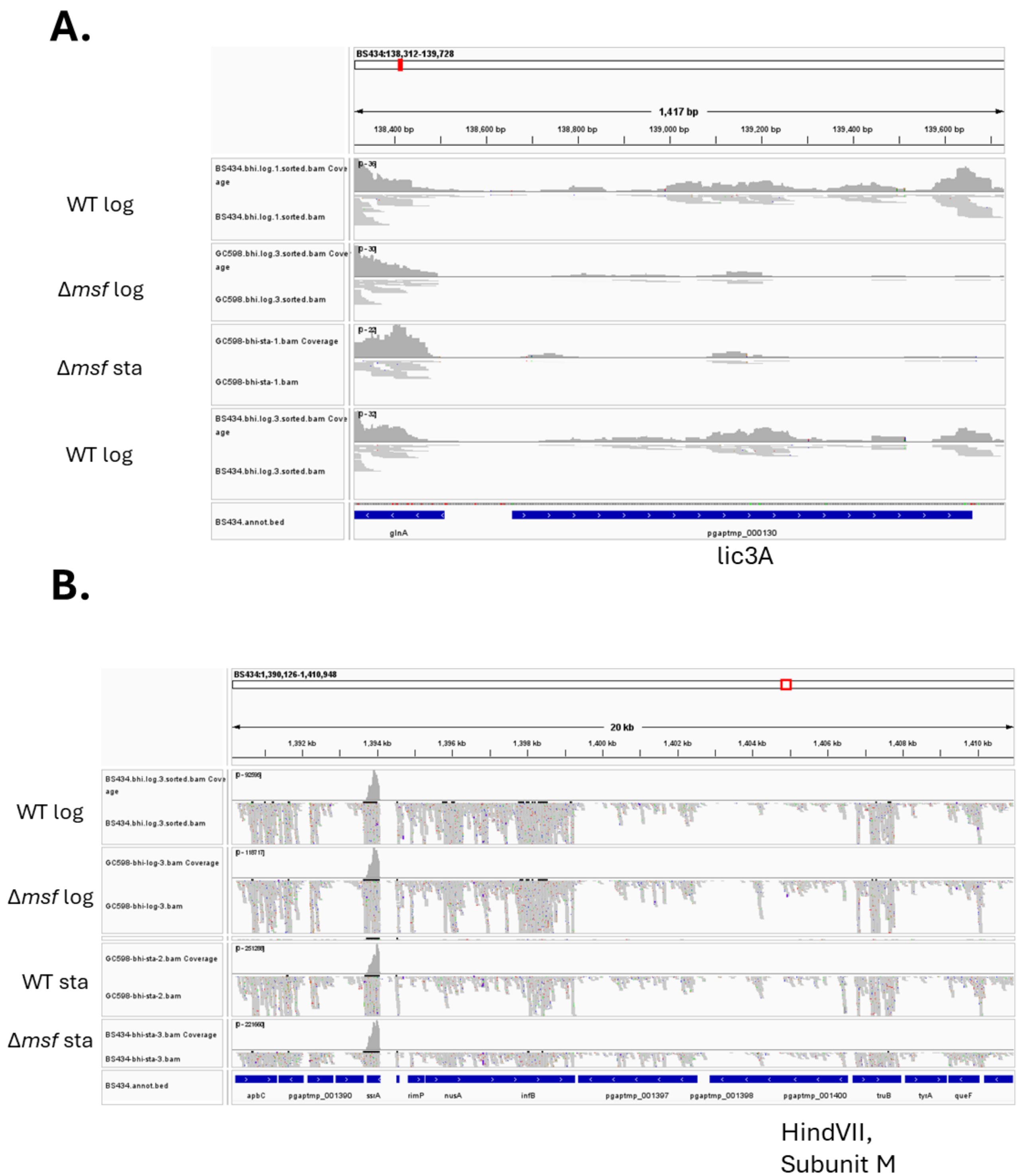

Figure S6 – No detectable phase variation at lic3A or HindVII methylase between genotypes.

Repeats were identified within the nucleotide sequences of gene promoter regions. RNA-seq

reads were aligned to the reference genome, and transcript coverage across these repeat-containing regions was visualized using IGV for A. lic3A (pgaptmp_001607) and B. HindVII. (pgaptmp_001400). No differences were seen in reads mapping in the promoter region, suggesting phase-variation status is the same between the wild type and mutant.

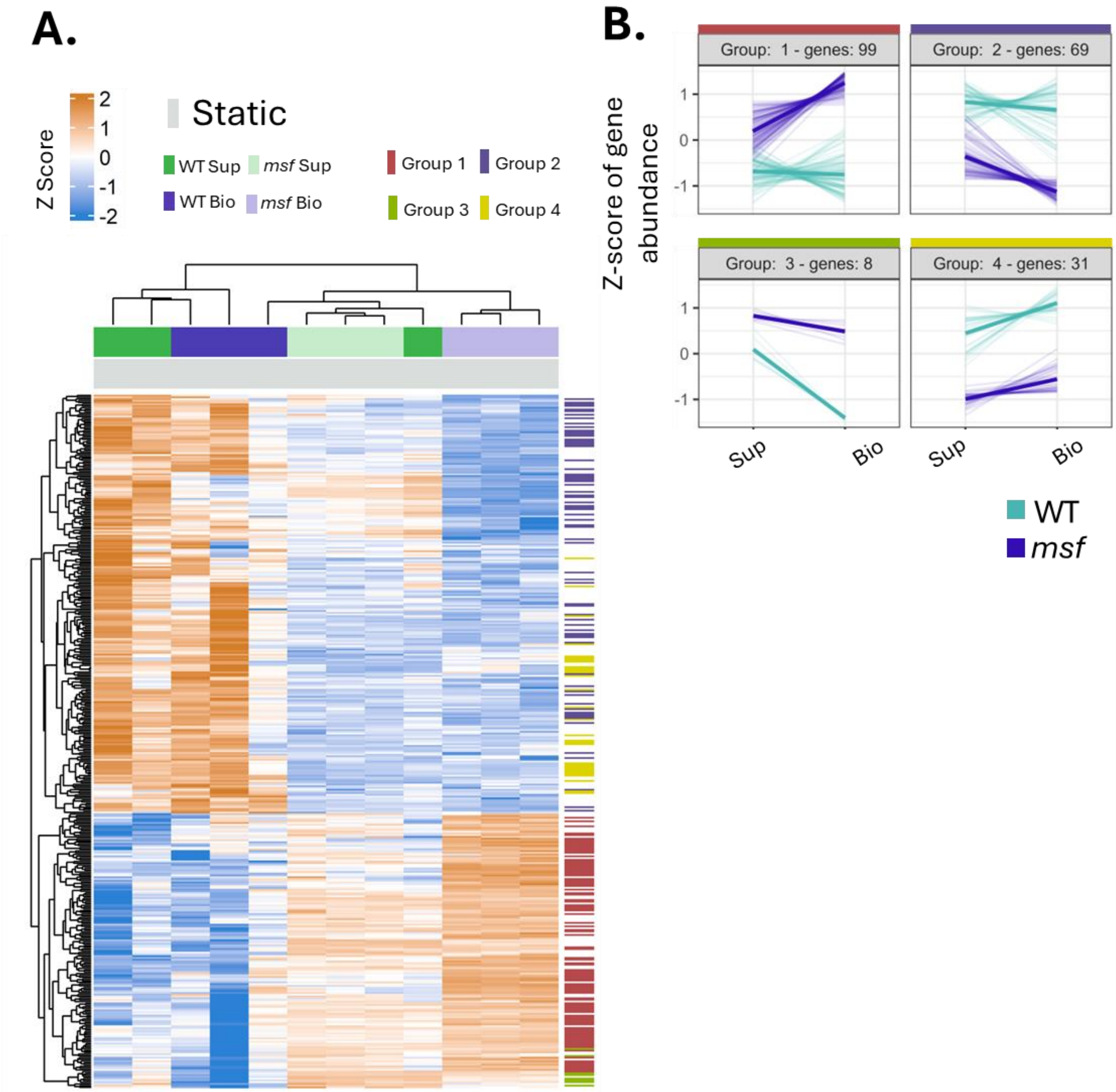

Figure S7 – The effect of msf on gene expression in static cultures. A. Heatmap of Z-scored gene expression values across static conditions for wild-type and mutant strains. Genes with similar patterns of expression were clustered using DEGPatterns, identifying four major gene groups, indicated by consistent color bars along the right side of the heatmap. Colors represent

the same cluster assignments shown in panel B. B. Smoothed expression patterns for each gene cluster, plotted as Z-scores of gene abundance across static conditions. Lines represent individual genes, and bold traces indicate mean expression patterns for wild-type and msf backgrounds.

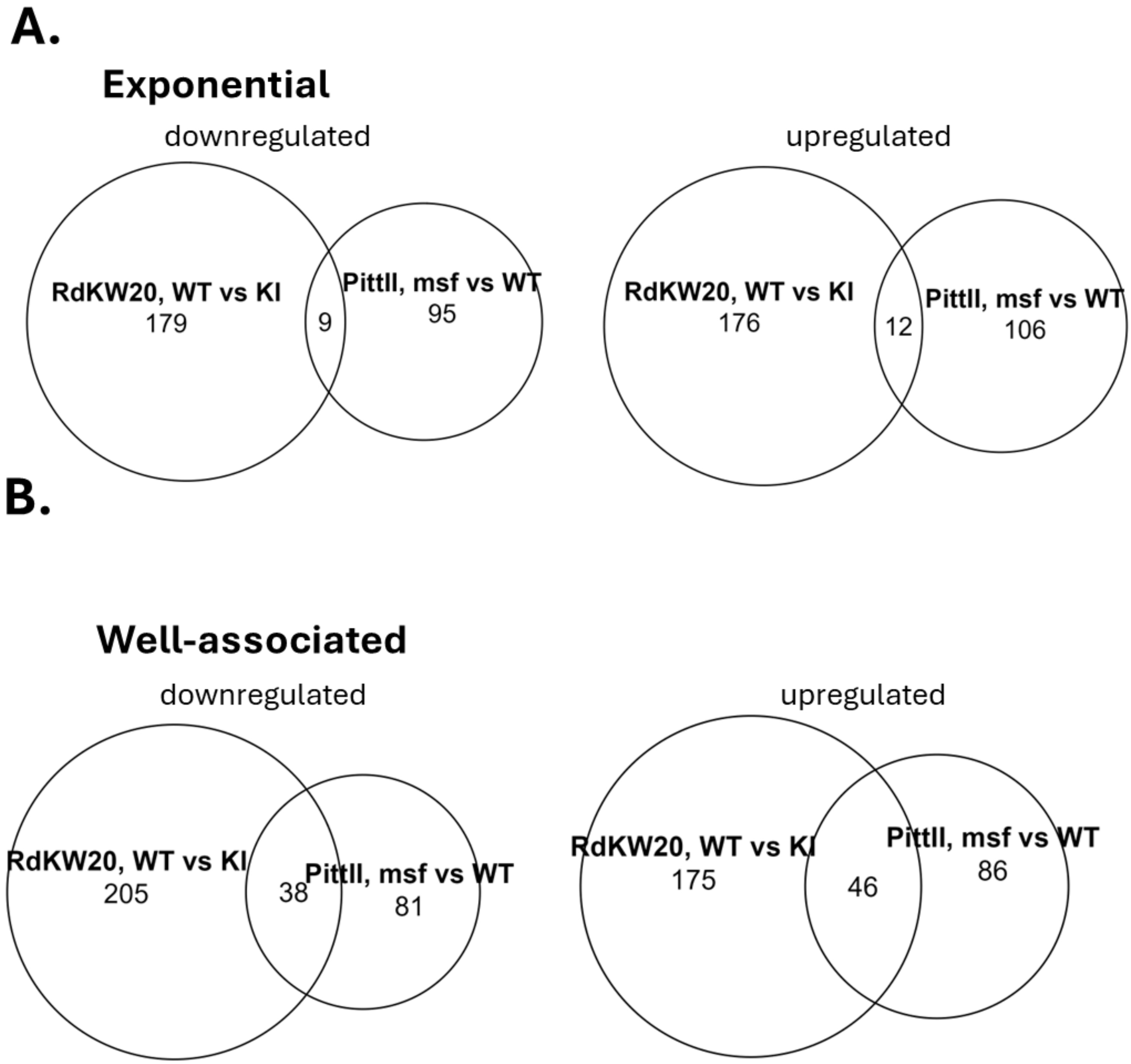

Figure S8 – Venn diagram overlaps between PittII and Rd DEGs. Lists of differentially expressed genes (DEGs) from the two different transcriptome experiments were compared in order to determine overlaps. The tests for both strains were performed in the same direction (msf minus vs msf plus) to better understand the role of msf. DEGs were considered significant with an adjusted p-value (Benjamini-Hochberg) of $p < 0.05$. No log (base 2) fold change cutoff was

included, but direction was determined. A. DEGs in exponential phase. B. DEGs in well-associated biofilms.

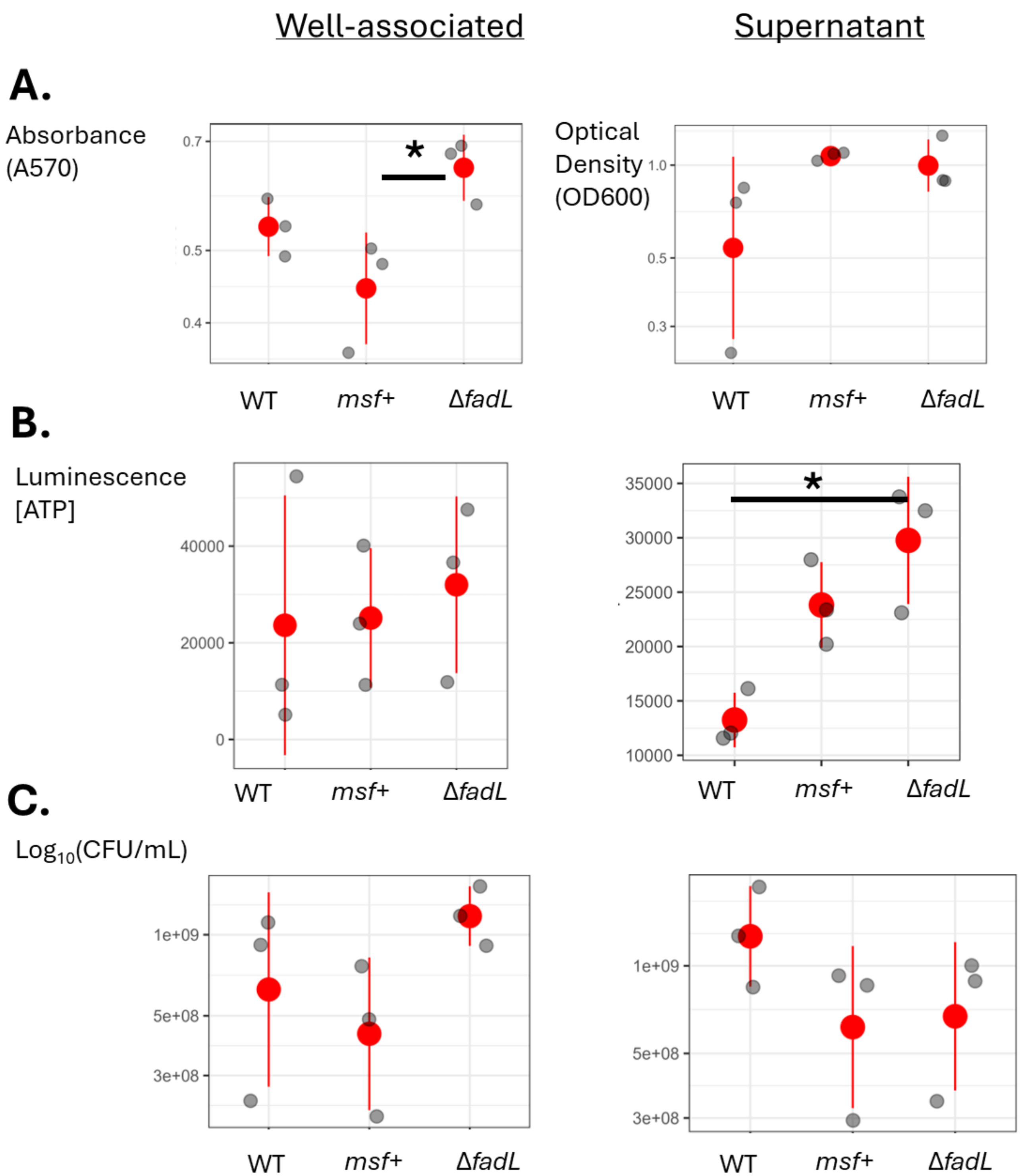

Figure S9 – Phenotypic assays Rd, knock-in, and OMPP1/fadL mutant. A. Crystal violet staining of wild type and mutant 24-hour static culture biofilms and OD600 measurements of 24-hour

supernatant cultures. B. Luminescence proportional to the concentration of ATP in the cultures. C. CFU/mL of well-associated biofilm and supernatant cultures grown in static conditions for 24 hours. One-way ANOVA was used to determine significant differences between genotypes for each assay and fraction and the Tukeys HSD test was used to identify which pair of means were significantly different (A. msf+ and fadL: $p = 0.02377 < 0.05$, B. WT and fadL: $p = 0.008$); CFU/ml measurements were log-transformed before statistical testing.
