# Supplementary material for: The msf gene causes condition-specific shifts in global gene expression in Haemophilus influenzae
Source: mBio. 2026 Jul 29;17(9):e01263-26. doi: 10.1128/mbio.01263-26 (PMC13556256; doi:10.1128/mbio.01263-26)
Supplement: Supplementary file 2 [file mbio.01263-26-s0002.pdf]

Supplementary Table Captions

Table S1 – Full Y2H prey fragment results. Table of the resulting 284 in-frame prey fragments which resulted from the Hybrigenics Y2H screen. For each Clone.Name, the fragment theoretical sequence was blasted against a database of clustered H. influenzae protein-coding sequences to obtain the Roary cluster ID. The resulting bitscore and query coverage were used to identify the best hit for each prey fragments which was fully contained within a single gene cluster.

Table S2 – Full Y2H summary without qcov filter. Table of the full 295 prey results without a query coverage filter summarized to show the total number of clones which received a predicted biological score, "PBS", of A through F. The Roary cluster ID and annotation are included along with notes which suggest cellular localization.

Table S3 – RNA-Seq Metrics. Sequencing metrics and sample meta data for the two RNA-seq experiments performed. Sample ID is the full name of the sequenced library. Sequenced reads were aligned to the respective reference genome using BWA on default settings and reads were sorted using Samtools. Total and mapped reads were determined using Samtools Flagstats.

Table S4 – RNA-Seq Count Table with Annotation; PittII. Full count table for the PittII RNA-seq experiment with BED formatted annotations. If available, the genes name, COG category, and COG description are noted.

Table S5 – Full Pairwise DEG Counts. Results of the number of differentially expressed genes for each pairwise comparison. Each comparison consists of the Wald Test performed between the set of samples defined by 'treatment' and the 'control' for both genotypes (top: condition), and between genotypes for all conditions (bottom: genotype). DEGs is the total number of DEGs;

NAs represents the number of genes which did not receive a p value due to genes with outlier counts or having low mean expression values. Columns Up and Down >2-fold are the sums of the genes with a log2fold change of $> |1|$.

Table S6 – Anaerobic and exponential-CDM DEGs. The DEG results of the pairwise Wald tests with wild-type controls and msf as treatment for early and late anaerobic exponential, and CDM exponential conditions. For each condition, the DEGs with a p adjusted < 0.05 (Benjamini-Hochberg procedure) are reported in addition to their log (base 2) fold change. If known, the gene name, Panaroo cluster, COG category, and annotation are reported.

Table S7 – GSEA core enrichment results. The log fold changes of all 1960 genes were used in a gene set enrichment analysis (GSEA) to identify if any COG categories were significantly enriched. For each COG category, the description, COG ID, and functional pathway are noted. The normalized enrichment score quantifies how much a gene set is enriched, and the adjusted pvalue for the COG category is included. The genes which define the "leading edge" of the core enrichment (ex. the genes with the drive the enrichment of a category in a given direction) were identified and their log fold changes from the pairwise Wald tests were included.

Table S8 – Shaking culture DEGs from condition LRT. The 81 DEGs identified as significant due to the loss of msf across the two shaking culture conditions in sBHI, exponential and stationary phase. The DEGs were clustered with DEGPatterns to identify similar patterns of expression, and if present, the gene's cluster is noted. The cluster color (green for cluster 1, blue for cluster 2) match the colored bar on the heatmap of Figure 3B and the bars above Figure 3C. If available, the gene name, COG category, COG description, COG ID, and functional pathway are annotated.

Table S9 – Static culture DEGs from condition LRT. The 396 DEGs identified as significant due to the loss of msf across both 24-hour fractions. The DEGs were clustered with DEGPatterns to identify similar patterns of expression, and if present, the gene's cluster is noted. The cluster color (green for cluster 1, blue for cluster 2, magenta for cluster 3, gold for cluster 4) match the colored bar on the heatmap of Figure S6A and the bars above Figure S6B. If available, the gene name, COG category, COG description, COG ID, and functional pathway are annotated.

Table S10 – Supernatant DEGs with Venn diagram overlap. The 48 DEGs identified between the pairwise comparison of msf and WT for the 24-hour supernatant fraction. The intersect column defines which DEGs were also present in the well-associated results, and if so, the direction of the log (base 2) fold change. If present, the gene name, COG category, COG description, and functional pathway are noted.

Table S11 – Static culture DEGs from interaction LRT. A likelihood ratio test (LRT) was performed using read counts from the 24-hour supernatant and well-associated conditions to identify a genotype by environment interaction. All significant DEGS ($p< 0.05$) were clustered using DEGPatterns and genes with similar expression patterns were clustered together. If available, the gene name, COG category, COG description, and COG functional pathway are noted.

Table S12 – RNA-seq Count Table with Annotation; Rd KW20. Full count table for the RdKW20 and msf knock-in RNA-seq experiment with BED formatted annotations. If available, the genes name, COG category, and COG description are noted.

Table S13 – Panaroo core gene cluster liftover table. Gene clusters produced via Panaroo for non-pseudogene protein-coding genes. The first column represents the core gene cluster defined

by the default parameter settings of Panaroo. If present, the locus tag for the strain, PittII or RdKW20 is noted.
